# Basal Forebrain Chemogenetic Inhibition Converts the Attentional Control Mode of Goal-Trackers to That of Sign-Trackers

**DOI:** 10.1523/ENEURO.0418-22.2022

**Published:** 2022-12-16

**Authors:** Aaron Kucinski, Cassandra Avila, Martin Sarter

**Affiliations:** Department of Psychology, University of Michigan, Ann Arbor, 48109, MI

**Keywords:** addiction, attention, basal forebrain, DREADD, rats, sign-tracking

## Abstract

Sign tracking versus goal tracking in rats indicate vulnerability and resistance, respectively, to Pavlovian cue-evoked addictive drug taking and relapse. Here, we tested hypotheses predicting that the opponent cognitive-behavioral styles indexed by sign tracking versus goal tracking include variations in attentional performance which differentially depend on basal forebrain projection systems. Pavlovian Conditioned Approach (PCA) testing was used to identify male and female sign-trackers (STs) and goal-trackers (GTs), as well as rats with an intermediate phenotype (INTs). Upon reaching asymptotic performance in an operant task requiring the detection of visual signals (hits) as well as the reporting of signal absence for 40 min per session, GTs scored more hits than STs, and hit rates across all phenotypes correlated with PCA scores. STs missed relatively more signals than GTs specifically during the last 15 min of a session. Chemogenetic inhibition of the basal forebrain decreased hit rates in GTs but was without effect in STs. Moreover, the decrease in hits in GTs manifested solely during the last 15 min of a session. Transfection efficacy in the horizontal limb of the diagonal band (HDB), but not substantia innominate (SI) or nucleus basalis of Meynert (nbM), predicted the behavioral efficacy of chemogenetic inhibition in GTs. Furthermore, the total subregional transfection space, not transfection of just cholinergic neurons, correlated with performance effects. These results indicate that the cognitive-behavioral phenotype indexed by goal tracking, but not sign tracking, depends on activation of the basal forebrain-frontal cortical projection system and associated biases toward top-down or model-based performance.

## Significance Statement

Sign-tracking rats (STs) have emerged as a model to study the neuro-behavioral mechanisms which bestow vulnerability for addiction-like behavior. The trait indexed by sign-tracking includes a bias toward bottom-up, or cue-driven attention, and has been hypothesized to be mediated in part by a low-capacity forebrain cholinergic system. Here, we show that compared with their counterparts, the goal trackers (GTs), the attentional performance of STs declines over prolonged time on task. Chemogenetic inhibition of the basal forebrain rendered the attentional performance of GTs to be similar to that of STs while not affecting the latter phenotype. Insufficient recruitment of the basal forebrain-cortical projection systems contributes to the addiction vulnerability-predicting trait indexed by sign tracking.

## Introduction

Structural irregularities in cortical and subcortical regions, and associated impairments in attentional control, impulsivity and compulsivity have been observed in persons with substance use disorder. These abnormalities may represent preexisting biopsychological risk factors and not result from drug taking per se, although drug use may further impair cortico-subcortical interactions (Volkow et al., 2005; Tomasi et al., 2007; Ersche et al., 2010, 2011, 2012; Goldstein and Volkow, 2011; Robbins et al., 2012).

Sign tracking is a behavioral predictor of a propensity for drug-taking and relapse (Saunders and Robinson, 2010; Meyer et al., 2012; Robinson et al., 2014). Sign-tracking rats (STs) are screened from outbred populations based on their propensity to approach and contact a Pavlovian food cue [Pavlovian Conditioned Approach (PCA) test]. Their counterparts, the goal-tracking rats (GTs), acquire the informational value of such a cue, the prediction of food delivery, but they do not approach and manipulate the Pavlovian cue. Compared with GTs, STs not only approach reward cues but also work more avidly to gain access to such cues, and such cues are more effective in instigating reward-seeking behavior in STs than GTs (Flagel et al., 2007, 2009; Saunders and Robinson, 2010; Robinson et al., 2014).

Attentional control deficits have been considered an essential component of psychological traits associated with addiction vulnerability (Black et al., 2006; Tomasi et al., 2007; Field and Cox, 2008; Marhe et al., 2013; Kilts et al., 2014). The propensity of STs to approach and contact Pavlovian reward cues may reflect in part a relative weakness of their attentional control, revealing their bias toward bottom-up, or signal-driven attention. In contrast to STs, GTs were demonstrated to attend to complex discriminative cues predicting the availability of addictive drugs, and they integrate higher-order contextual information with the enhanced motivational state evoked by such cues (Pitchers et al., 2017a).

Consistent with the considerable evidence indicating an essential role of the basal forebrain cholinergic projection system to cortex in attention (for review, see Ballinger et al., 2016; Decker and Duncan, 2020; Sarter and Lustig, 2020), GTs, but not STs, activate their cholinergic system in the presence of a Pavlovian drug cue (Pitchers et al., 2017b), and cholinergic lesions abolish the ability of GTs to respond to complex occasion-setting cues (Pitchers et al., 2017a). In STs, the limited cholinergic responses to conditioned drug cues and attentional performance (Paolone et al., 2013) have been demonstrated to be related to the failure of intracellular neuronal choline transporters (CHT) to populate the synaptosomal plasma membrane (Koshy Cherian et al., 2017).

Here, we first wished to determine the precise behavioral characteristics of the relatively poor attentional control that has been hypothesized to constitute an essential variable of the cognitive-behavioral trait indexed by sign tracking. Second, given the hypothesis that such characteristics are mediated via a “dampened” basal forebrain projection system, we expected that chemogenetic inhibition of the basal forebrain further increases the severity of the expression of relatively poor attentional control in STs, while rendering the relatively superior attentional performance of GTs to resemble that of STs.

To assess the attentional performance of rats, we employed an operant sustained attention task (SAT) that required rats to execute discrete responses for reporting the presence or absence of a brief visual signal (McGaughy and Sarter, 1995; McGaughy et al., 1996; Echevarria et al., 2005; Luck et al., 2012). A relatively rich basis of evidence describes SAT performance of mice and rats, healthy humans, children, and patient groups (Demeter et al., 2008, 2013; Lustig et al., 2013; Berry et al., 2014, 2015, 2017; Sarter et al., 2016; Kim et al., 2019; Prasad and Lustig, 2020) and has informed the analysis of the psychological mechanisms underlying SAT performance (Chebolu et al., 2022).

Basal forebrain cholinergic and, to a relatively greater extent, noncholinergic projections form an anatomically heterogenous (Duque et al., 2000; Manns et al., 2000; Gritti et al., 2006; Hassani et al., 2009) yet functionally integrated projection system (Lin et al., 2006; Lin and Nicolelis, 2008; Tingley et al., 2015; Yang et al., 2017). Therefore, in the present experiment, chemogenetic inhibition targeted all neurons within the main subdivisions of the basal forebrain which give rise to projections to the cortex [nucleus basalis of Meynert (nbM); substantia innominate (SI); horizontal limb of the diagonal band (HDB)]. Immuno-histochemical analyses were designed to identify the potentially differential contributions of cholinergic versus noncholinergic neurons to the effects of chemogenetic inhibition.

## Materials and Methods

### Subjects and housing

Adult male and female Sprague Dawley rats (*N* = 106; 55 females) were purchased from Envigo. Animals were between two and three months of age on arrival. Rats first underwent Pavlovian Conditioned Approach (PCA) screening that yielded 46 GTs, 25 STs, and 35 INTs. Of the 51 male rats screened, there were 27 GTs, 6 STs, and 18 INTs, and of the 55 females, there were 19 GTs, 19 STs, and 17 INTs. Rats then underwent training on the Sustained Attention Task (SAT), followed by surgeries, resumption of SAT practice, and the assessment of effects of chemogenetic inactivation of the basal forebrain. Rats were approximately three months of age during PCA screening and between three and eight months of age during SAT training and testing.

Animals were individually housed in opaque single standard cages (27.70 × 20.30 cm) in a temperature-controlled and humidity-controlled environment (23°C, 45%) and maintained under a 12/12 h light/dark schedule (lights on at 7:00 A.M.). Food (Envigo Teklad rodent diet) and water were available *ad libitum* during PCA screening and recovery from surgeries. Water was progressively restricted (to 12 h, 8 h, 4 h, 2 h, 1 h, and 30 min free water/d) in the week before the start of SAT training. Rats received 30 min of free water on days which they did not undergo SAT testing and 15 min of free water following completion of SAT sessions, in addition to water rewards received during the task. PCA and SAT testing were conducted during the light phase (7:00 A.M. to 7:00 P.M.). At the onset of PCA testing, animals’ body weights did not differ between subsequently determined phenotypes (*F*_(2,44)_ = 1.01, *p *=* *0.37; M ± SD: 321.78 ± 9.61 g). Likewise, body weights also did not differ between phenotypes during the assessment of baseline SAT performance (*F*_(2,44)_ = 0.85, *p *=* *0.43; 337.22 ± 9.81 g) nor during the period when effects of CNO were determined (*F*_(2,44)_ = 1.00, *p *=* *0.38; 350.64 ± 8.98 g).

All procedures were conducted in adherence with protocols approved by the Institutional Animal Care and Use Committee of the University of Michigan and in laboratories accredited by the Association for Assessment and Accreditation of Laboratory Animal Care.

### PCA screening

#### Apparatus and procedures

**Figure 1.**
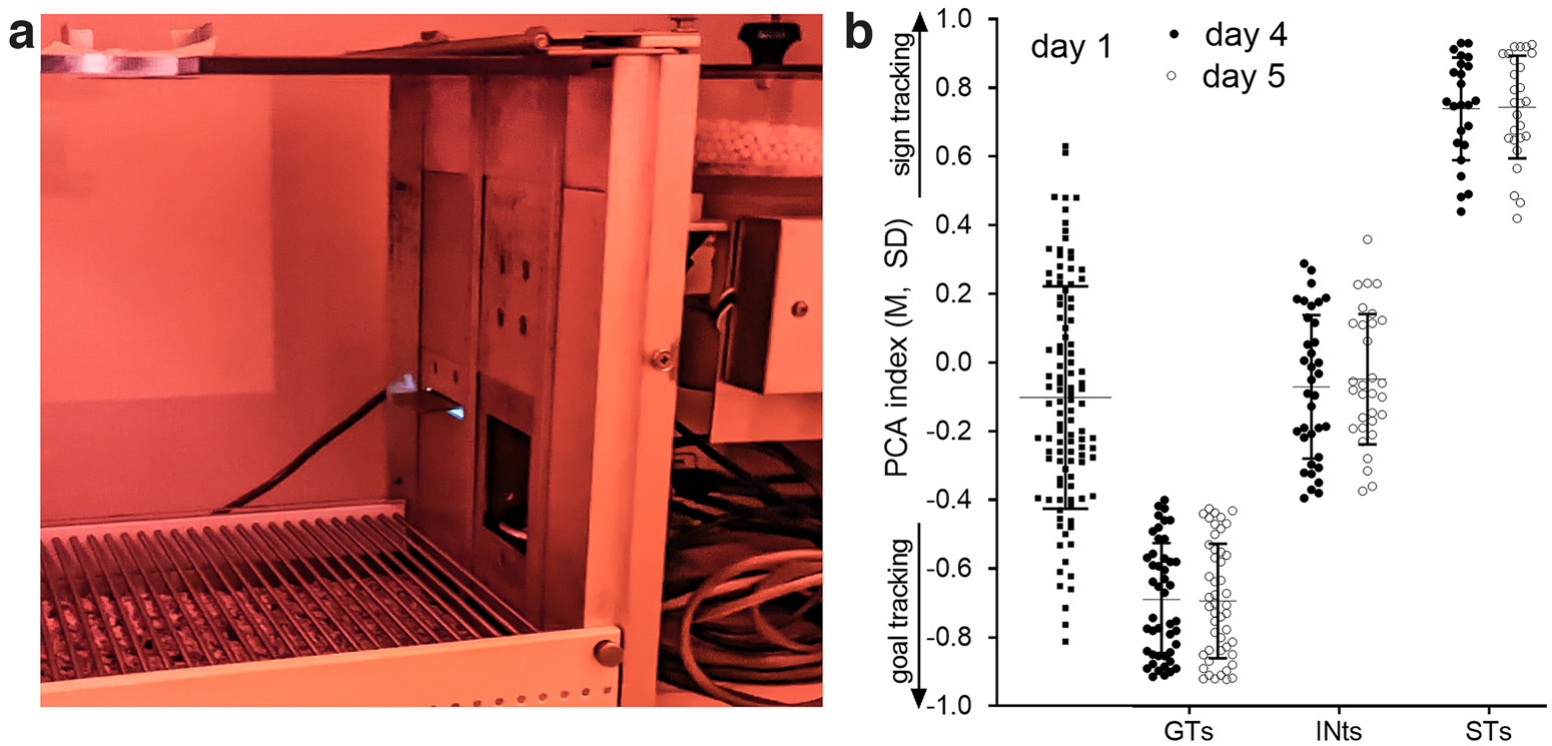
Depiction of the apparatus used to assess Pavlovian Conditioned Approach (PCA) behavior (***a***) and determination of ST, GT, and INT phenotypes [***b***; *N* = 106, 55 females; shown are individual data points, means and SD (M ± SD)]. PCA behavior was assessed across five consecutive sessions/days. ***a***, Illumination of a red house light indicated the beginning of a PCA test session. The CS for each trial was the extension of the illuminated lever into the chamber for 8 s (shown on the left of the chamber’s intelligence panel). After lever retraction, a pellet was immediately delivered into the magazine (bottom of the center of the panel). Rats were presented with 25 lever/pellet pairings delivered on a VI-90 (30–150 s) schedule. On average, PCA test sessions lasted 37.5 min. ***b***, Positive PCA scores indicate a bias toward approach and operation of the Pavlovian food cue (STs), while negative PCA scores indicate a bias toward approaching the magazine (GTs). Dashed lines indicate the ±0.4 cutoff score for assigning the three phenotypes. Scores obtained from the first session (left) indicated that the majority of rats did not exhibit a bias toward the lever (CS) or the magazine. By days 4 and 5 (right), nearly a third of the rats had developed a preference for approaching the CS while 46/106 rats emerged as goal-trackers (the final classification was based on the average of individual PCA scores from sessions 4 and 5). In this sample, females were more likely to be classified as STs and males were more likely to emerge as GTs (see Results).

The screening of STs and GTs using a PCA test followed established and previously described methods (Lovic et al., 2011; Paolone et al., 2013; Yager et al., 2015; Pitchers et al., 2017a; Campus et al., 2019; Phillips and Sarter, 2020). Briefly, rats were handled daily for 3 d and given ∼15 banana-flavored grain-based pellets (45 mg; BioServ) in their home cages for 2 d before the start of PCA testing. Rats were tested in conditioning chambers (20.5 × 24.1 cm floor area, 20.2 cm high; MED Associates Inc.). Throughout the duration of the experiments, males and females were tested in separate chambers. A food magazine with an automatic feeder, delivering banana pellets, was located in the center of one of the walls of the chamber. Infrared photobeam breaks detected magazine entries. On either the left or the right side of the magazine was a retractable lever with an LED backlight that was illuminated only when the lever extended into the chamber (Fig. 1*a*). Deflections of the lever were used to quantify lever contacts. The beginning of a test session was signaled by the illumination of a red house light located near the ceiling of the side of the chamber opposite to the magazine/lever. On the first day of testing (“pretraining”), rats were placed into the conditioning chambers and the house light was illuminated after a 5-min habituation period. Twenty-five pellets were then delivered on a VI-30 (0–60 s) schedule. On average, this pretraining session lasted 12.5 min, and the lever was retracted throughout the session. During this session and all subsequent PCA sessions, rats consumed all pellets. The house light was turned on in the next five PCA sessions and rats were presented with 25 lever/pellet pairings delivered on a VI-90 (30–150 s) schedule. The CS for each trial was the extension of the illuminated lever into the chamber for 8 s. After lever retraction, a pellet was immediately delivered into the magazine. On average, the PCA test sessions lasted 37.5 min.

#### PCA measures and classification criteria

Lever presses and magazine entries during the CS periods were used to quantify three measures of approach that determined the PCA index score. (1) Response bias was defined as the difference between lever presses and magazine entries, expressed as a proportion of the total responses [(lever presses − magazine entries)/(lever presses + magazine entries)]. This value yielded a score between −1.0 and 1.0, with positive values indicating a bias toward pressing the lever (ST) and negative values a bias toward entering the magazine (GT) during CS presentation (lever extension before the delivery of food rewards). (2) Latency score was calculated as the difference between the latency to approach the lever and the magazine after CS presentation; this difference was normalized by dividing by the maximum 8-s latency [(magazine latency − lever latency)/8]. Negative values indicated rats that rapidly approached the magazine (GT) and positive values indicated that rats quickly approached the lever during CS presentation (scores again were between −1.0 and 1.0). (3) Probability difference was calculated as the difference between the probabilities of pressing the lever during the CS (i.e., the number of trials with a lever press out of 25 trials) minus the probability of entering the magazine. For this value, GTs approached −1.0 and STs approached 1.0. The final “PCA index score” represented the average of the three values described above: response bias score, latency score, and probability difference. The values of this score also ranged from 1.0 to −1.0, with a score of 1.0 indicating approaches and contacts of the lever on every trial, and a score of −1.0 indicating approaches and contacts of the magazine entry on every trial. Rats with an averaged PCA index score from PCA sessions 4 and 5 ranging from −1.0 to −0.4 were defined as GTs (i.e., rats more likely to direct behavior toward the food magazine than the lever), and rats with a PCA index score between +0.4 and +1.0 were designated as STs (i.e., rats more likely to direct behavior toward the lever-CS than the food magazine). Rats with scores ranging from −0.39 to +0.39, whose behavior vacillated between lever-CS and food magazine, were classified as INTs. Approach responses (response probability, number of contacts, and latency) were analyzed with repeated-measures ANOVAs with phenotype (STs, GTs) as the between-groups measure and training day as the within-subject factor.

### SAT

#### Apparatus

Training and testing were conducted using 12 operant chambers (MED Associates Inc.) housed within individual sound-attenuating cubicles. Males and females were tested in separate chambers. Each chamber was equipped with two retractable levers, a central panel white light (2.8 W), and a water dispenser located on the same wall as the panel lights. The water dispenser delivered 45 μl of water per correct response. Signal presentation, lever operation, reinforcement delivery, and data collection were controlled by a Pentium PC and Med-PC for Windows software (version 4.1.3; MED Associates).

#### Acquisition

Water-deprived rats (one week of a progressively reduced period of free water, down to 30 min on the final day) were initially trained to press a lever for a water reward in accordance with a modified fixed ratio-1 (FR1) schedule for water reinforcement. Two levers were extended throughout the session (to the left and right of the water port). During this training phase, any lever press resulted in water delivery. Typically, the animals did not exhibit a side bias with regard to which lever is pressed; however, if one lever was pressed five times in succession, the FR1 schedule was modified to require the animal to press the opposite lever before the next reward could be obtained. After three consecutive days with 120 reinforced lever presses under 20 min, the rats began training to discriminate between a signal (1-s illumination of the central panel light) and a nonsignal (no illumination) event (for luminance measures see Phillips and Sarter, 2020). Two seconds after a signal or nonsignal event, both levers were extended into the operant chamber and remain extended for 4 s or until a lever was pressed. If no press occurred after 4 s, the levers were retracted and an omission was recorded. Immediately following responses (either correct or incorrect), both levers were retracted and the variable intertrial interval (ITI; 12 ± 3 s) was reset. On signal trials, a press of the left lever was reinforced and termed a “hit,” whereas a press of the right lever was not reinforced and termed a “miss.” On nonsignal trials, a press of the right lever was reinforced and termed a “correct rejection,” whereas a press of the left lever was not reinforced and termed a “false alarm.” Animals received water rewards only for correct responses (45 μl for each hit and correct rejection), whereas incorrect responses (misses and false alarms) were not rewarded. Half of the animals were trained with the opposite pattern (boxes 1–12, alternating) to eliminate the possibility of a selection bias. Signal and nonsignal events were presented in pseudo-random order for 81 trials each (total of 162 trials) per session. During this training phase, incorrect responses were followed by correction trials in which the previous trial was repeated. After three consecutive incorrect responses on correction trials, the animal underwent a forced trial in which the lever was extended for 90 s or until the animal made a response. If the forced-choice trial was a signal trial, the signal light remained illuminated for as long as the lever was extended. The house light was not illuminated during this training stage.

Animals progressed to the subsequent step of shaping if they responded correctly to ≥70% of both signal and nonsignal trials for three consecutive days. During the third phase of shaping, multiple signal durations (500, 50, and 25 ms) were introduced and the ITI was reduced to 9 ± 3 s. Correction and forced-choice trials were also eliminated. Trial type and signal duration were pseudo-randomly determined for each trial. Session length was set at 42 min (including a 3-min habituation period on the start of each session). After stable performance was achieved, defined by at least 70% hits to 500-ms signals, 70% correct rejections, and ≤30% omissions, animals began training in the final version of the task. The final version was identical to the previous training stage, except that the house light was illuminated throughout the session. The addition of the illuminated house light represents a crucial element of testing sustained attention as it requires the animal to constrain its behavior and focus on the central panel light during task performance. Upon reaching the final stage of training before lesion surgeries, animals remained at this stage until they reached a stable performance of at least 60% hits to 500-ms signals, 70% correct rejections, and ≤30% omissions for three consecutive sessions.

#### Measures of SAT performance

The following behavior measures were recorded during each SAT session: hits, misses, false alarms, correct rejections, and omissions. Misses and false alarms are the inverses of hits and correct rejections, respectively. The relative number of hits (hits/hits + misses) for each signal length as well as the relative number of correct rejections (correct rejections/correct rejections + false alarms) were calculated. Response bias (*B˝_D_*) was determined based on the proportion of hits (HP) and proportion of false alarms (FAP). Using performance with the 500-ms duration signal, *B˝_D_* was calculated using the formula: *B˝_D_* = [(1 – HP)(1 – FAP) – HP FAP]/[(1 – HP)(1 – FAP) + HP FAP] (Donaldson, 1992). *B˝_D_* values range from −1 to +1, with negative values indicating a (liberal) bias toward reporting the presentation of a signal, and positive values indicating a (conservative) bias toward reporting the absence of a signal. A *B˝_D_* value of 0 indicates no bias (for a prior analysis of these parameters based on data from humans performing the SAT, see Demeter et al., 2008).

#### Postsurgery performance and effects of CNO

Following surgeries and postsurgery SAT practice, the effects of CNO were assessed. Rats were administered either vehicle or CNO (pseudo-random assignment) and, 5–7 d later, CNO or vehicle.

CNO was obtained from Tocris Bioscience and dissolved in 10 mg/ml in 6% DMSO in 0.9% NaCl solution. Injections of CNO or vehicle (intraperitoneal) were given at a dose of 5.0 mg/kg 15–20 min before SAT testing. We previously did not find effects of this dose of CNO in control construct-expressing rats performing an attention-demanding motor task (Kucinski et al., 2019). This finding was consistent with the proposed conversion rate of CNO to clozapine (Gomez et al., 2017), yielding an equivalent clozapine dose of 0.05 mg/kg. However, a 50- to 100-fold higher dose of clozapine is required to produce direct effects on brain neurochemistry and trained behaviors (Parada et al., 1997; Gemperle et al., 2003; Rueter et al., 2004; Martinez and Sarter, 2008).

### Designer Receptors Exclusively Activated by Designer Drugs (DREADD) virus expression

About two-thirds of basal forebrain cholinergic neurons are noncholinergic and predominantly GABAergic (Gritti et al., 2006). Because basal forebrain cholinergic and noncholinergic neurons mediate overlapping functions, including the generation of high-frequency oscillations in the cortex (Lin et al., 2006; Lin and Nicolelis, 2008; Gritton et al., 2016; Howe et al., 2017), the necessity of basal forebrain neuronal activity for the relatively superior SAT performance of GTs was tested by nonselectively expressing an inhibitory DREADD in cholinergic and noncholinergic neurons. The viral vector containing plasmid pAAV-hSyn-hM4D(Gi)-mCherry was obtained from AddGene (AddGene plasmid #50 475-AAV8). The adeno-associated virus (AAV), containing a double floxed muscarinic Gi-coupled-receptor, hM4D (Armbruster et al., 2007; Roth, 2016), fused with mCherry and under the control of human synapsin promoter, was stereotaxically infused into the basal forebrain, targeting primarily the fronto-medial cortical projection systems arising from the HDB. Rats were placed in vaporization chambers and anesthetized with 4–5% isoflurane delivered at 0.6 l/min O_2_ using a SurgiVet Isotec four Anesthesia Vaporizer until the animals were no longer responsive to a tail pinch and exhibited no hindlimb withdrawal reflex. Heads were shaved using electric clippers and cleaned with a betadine scrub and alcohol pad. The animals were then mounted to a stereotaxic instrument (David Kopf Instruments) and isoflurane anesthesia was maintained at 1–3% for the remainder of the surgery. An ∼2.5-cm incision was made down the midline of the scalp to expose the skull. The animals’ body temperature was maintained at 37°C using Deltaphase Isothermal Pads (Braintree Scientific). Ophthalmic ointment was used for eye lubrication. To prevent hypovolemia and hemodynamic instability, 1 ml/100 g 0.9% NaCl was administered subcutaneously. Animals also received an injection of an analgesic (carprofen; 5.0 mg/kg, s.c) before surgery and once or twice daily as necessary for 48 h postoperatively. One microliter of AAV-hSyn-hM4D(Gi)-mCherry vector was infused (bolus) into the basal forebrain at two sites per hemisphere (AP: −0.4; ML: ±2.4; DV: −7.6; AP: −0.8; ML: ±2.9; DV: −8.0 mm). The injector was left in place for 8 min to minimize diffusion into the injector tract.

#### Visualization and quantification of DREADD transfection space

Following the completion of SAT testing, rats were deeply anesthetized with a lethal dose of sodium pentobarbital (270 mg/kg, i.p.) and transcardially perfused with PBS, followed by 4% paraformaldehyde in 0.15 m sodium-phosphate solution, pH 7.4. Brains were extracted and postfixed in 4% paraformaldehyde for 24 h, rinsed with PBS and then placed in 30% sucrose solution until they sank. Brains were sectioned into 35-μm thick slices using a freezing microtome (CM 2000R; Leica) and stored in cryoprotectant until further histologic processing.

To determine the efficacy and selectivity of mCherry expression in BF cholinergic and noncholinergic neurons, sections were stained to amplify the signal of the mCherry fluorescent tag and to visualize cholinergic neurons, using antibodies against the vesicular acetylcholine (ACh) transporter (VAChT; Arvidsson et al., 1997). Sections were first rinsed six times for 5 min each in 0.1 m PBS, pH 7.3, then incubated in 0.1% Triton X-100 diluted in PBS for 15 min (Agostinelli et al., 2019). Sections were then rinsed with PBS three times for 5 min each and incubated with 1% normal donkey serum (NDS) and 1% Triton X-100 made in PBS for 60 min at room temperature (RT). The sections were incubated overnight in the primary antibodies (rabbit anti-mCherry: ab167453, Abcam; goat anti-VAChT: abn100; Millipore; 1:1000 for both). The following day, sections were rinsed three times for 5 min in PBS and then incubated in the secondary antibodies (donkey anti-rabbit conjugated to Alexa 594, A11058; ThermoFisher Scientific; donkey anti-goat conjugated to Alexa 488, A11055; ThermoFisher Scientific) for 90 min at room temperature. Sections were then incubated in DAPI (D21490; ThermoFisher Scientific; diluted to 300 nm in PBS) for 2 min at room temperature. All sections were rinsed three times for 5 min with PBS and then mounted, air dried, and cover-slipped with Vectashield Antifade Mounting Medium (H-1000; Vector Laboratories). A Zeiss LM 700 confocal microscope was used to inspect and photomicrograph fluorescent neurons at 10× (for cell count estimates), 20× (for verifying and documenting double-labeled cells), and 40× magnification at two A-P levels (−0.72 and −0.96 mm). The microscope was equipped for sequential multi-track acquisition with 405-, 488-, and 561-nm excitation lines and filter sets specific for DAPI (Zeiss filter set 49), Alexa 488 (Zeiss filter set 38 HE), and Alexa 594 (Zeiss filter set 54 HE), respectively. Single-labeled and double-labeled neurons were counted in three subregions of the basal forebrain [nucleus basalis of Meynert (nbM); horizontal limb of the diagonal band (HDB); substantia innominate (SI)]. Using the ImageJ multipoint tool, superimposed counting frames were generated for each subsection (nbM: 0.4 × 0.8 mm; HDB: 1.4 × 1 mm; SI: 0.5 × 0.9 mm) and counts were restricted to these frames. Counts were generated based on two sections per rat, yielding four counts per rat and subregion. For further analyses, averaged counts as well as the highest count among the four counts obtained from each brain and subregion were used. Tile scanning/stitching was used at the 10× (3 × 2 tiles, 3455.94 by 2370.64 μm) and 20× (4 × 3 tiles, 1407.88 by 1135.31 μm) magnifications. The resolution of the 20× images allowed for visualization of neuronal architecture, specifically fluorescence restricted to the soma versus puncta. The split view on Zen Black software was used to view single channels of the multi-track shots. Cells with green (Alexa 488) and red (Alexa 594) fluorescence located primarily in the soma, co-labeled with DAPI, were used in counts for double-labeled neurons.

To rank subregions in accordance with overall DREADD transfection efficacy, total mCherry-positive cell counts were categorized using a scale from 0 to 5 (0: 0 cells; 1: <20; 2: 20–30; 3: 31–60; 4: 61–80; 5: >80 cells). To quantify the portion of cholinergic neurons co-expressing the DREADD reporter fluorophore, the number of double-labeled neurons (mCherry-positive + VAChT-positive) was divided over the total number of mCherry-expressing neurons. To correlate transfection efficacy scores and the percent of double-labeled cells with the behavioral efficiency of DREADD activation in GTs, brain sections were processed and cell counts were generated from the five GTs that showed the largest decreases in hit rates following CNO administration when compared with their hit rates following vehicle injection (decreases ranging from 100.00% to 32.50%) as well as from the five GTs that exhibited the smallest effects of CNO (decreases ranging from 3.61% to an increase of 7.04%). Furthermore, and to test for the possibility that the relative lack of effect of CNO in STs was unrelated to the DREADD expression efficacy, we selected three STs with HDB cell counts that were within the range of cell counts of the five GTs that exhibited the largest CNO effects (reflecting the main finding in GTs) and compared the performance impact of CNO in these rats with those GTs.

### Statistical analyses

SAT performance measures (hits, correct rejections, and omissions) were analyzed primarily with mixed model repeated measures ANOVAs as well as one-way or two-way ANOVAs when applicable. The analyses of hits also included the within-subject factor signal duration (500, 50, and 25 ms). Following significant main effects, *post hoc* multiple comparisons were conducted using the least significant difference (LSD) test. Significant interactions between the effects of group and other factors were followed by one-way ANOVAs on the effects of group and LSD multiple comparison tests. α Was not corrected for *post hoc* multiple comparisons because these comparisons followed ANOVAs which already rejected the null hypothesis and therefore served solely to locate, and assist in the interpretation of, main effects and interactions (Rothman, 1990). For the analysis of effects of CNO on SAT performances, dose was used as a factor in the mixed model ANOVAs. χ^2^ and nonparametric statistical tests (detailed in Results) were employed to analyze the number of SAT blocks indicating above-chance performance levels. Spearman rank correlations coefficients (ρ) were used to determine relationships between the behavioral impact of CNO and the expression of the DREADD construct in cholinergic and noncholinergic neurons in three subregions of the basal forebrain.

#### Effects of sex

Approximately half of the rats used for these experiments were females. χ^2^ and nonparametric statistical tests (detailed in Results) were employed to analyze the PCA test-based classification of male and female rats per phenotype. Furthermore, the effects of sex on PCA scores from test day 5 were determined using an ANOVA on the effects of phenotype and sex. Furthermore, an initial ANOVA on the hit rate of STs, INTs, and GTs included the factor sex. For major results derived from parametric tests, effect sizes (Cohen’s *d*) were indicated (Cohen, 1988). Statistical analyses were performed using SPSS for Windows (version 17.0: SPSS). In cases of violation of the sphericity assumption, Huyhn–Feldt-corrected *F* values, along with uncorrected degrees of freedom, were given. α Was set at 0.05. Exact P values were reported as recommended previously (Greenwald et al., 1996; Sarter and Fritschy, 2008). Unless indicated otherwise, data graphs depict individual values, means and 95% confidence intervals (CIs).

## Results

### PCA testing and phenotype distribution by sex

A total of *N* = 106 rats (55 females) underwent five sessions/days of PCA testing. PCA scores from the 4^th^ and 5^th^ session were averaged for individual rats, and this average score determined phenotype assignment (GT, ST, INT). As in our previous experiments (Kucinski et al., 2019), we applied relatively liberal PCA cutoff scores to assign phenotypes (±0.4 as opposed to the more typical ±0.5); however, *post hoc* re-analyses indicated that applying the tighter cutoff scores did not significantly impact the results described below.

Figure 1*a* shows a photograph of the intelligence panel of the chambers used for PCA testing, with the CS extended and illuminated. Figure 1*b* depicts individual PCA scores obtained during the first, 4^th^ and 5^th^ PCA session. During the first session, most rats did not exhibit a preference for approaching and operating the lever (i.e., the CS) versus entering the magazine, as reflected by a mean PCA score of −0.10. Across subsequent testing sessions, an increasing number of rats developed a preference for the CS or the magazine, yielding a total of 46 GTs (19 females), 35 INTs (17 females), and 25 STs (19 females). A χ^2^ test of independence was performed to examine the relationship between sex and the manifestation of sign tracking versus goal tracking (χ^2^(2) = 8.04; *p *=* *0.02). This finding reflected that, in this sample, females were more likely to be classified as STs and males as GTs. However, such a relationship between these two categories was not reliably observed in prior experiments (Pitchers et al., 2015), perhaps reflecting considerable variations in the distribution of these phenotypes in cohorts obtained from different vendors, as well as from different breeding barriers of the same vendors, and associated genetic heterogeneity (Fitzpatrick et al., 2013; Gileta et al., 2022). Furthermore, an ANOVA on the effects of sex and phenotype on PCA scores obtained from test day 5 indicated neither a main effect of sex nor an interaction between the effects of sex and phenotype (both *p *>* *0.93). Thus, across the three phenotypes, sex did not influence the distribution of PCA scores, consistent with results from prior experiments (Pitchers et al., 2015; Peterson et al., 2020).

### STs scored fewer hits than GTs

The data from test sessions following administration of the vehicle for the DREADD agonist were used for an in-depth analysis of the attentional performance of the three phenotypes. Of the initial *N* = 106 rats that began SAT training, 51 rats (21 GTs, five females, 18 INTs, seven females, and 12 STs, six females) had previously reached SAT performance criterion and thus were included in this analysis. The proportion of rats reaching this stage did not depend on phenotype (χ^2^(2, *N* = 157) = 0.09; *p *=* *0.95).

**Figure 2.**
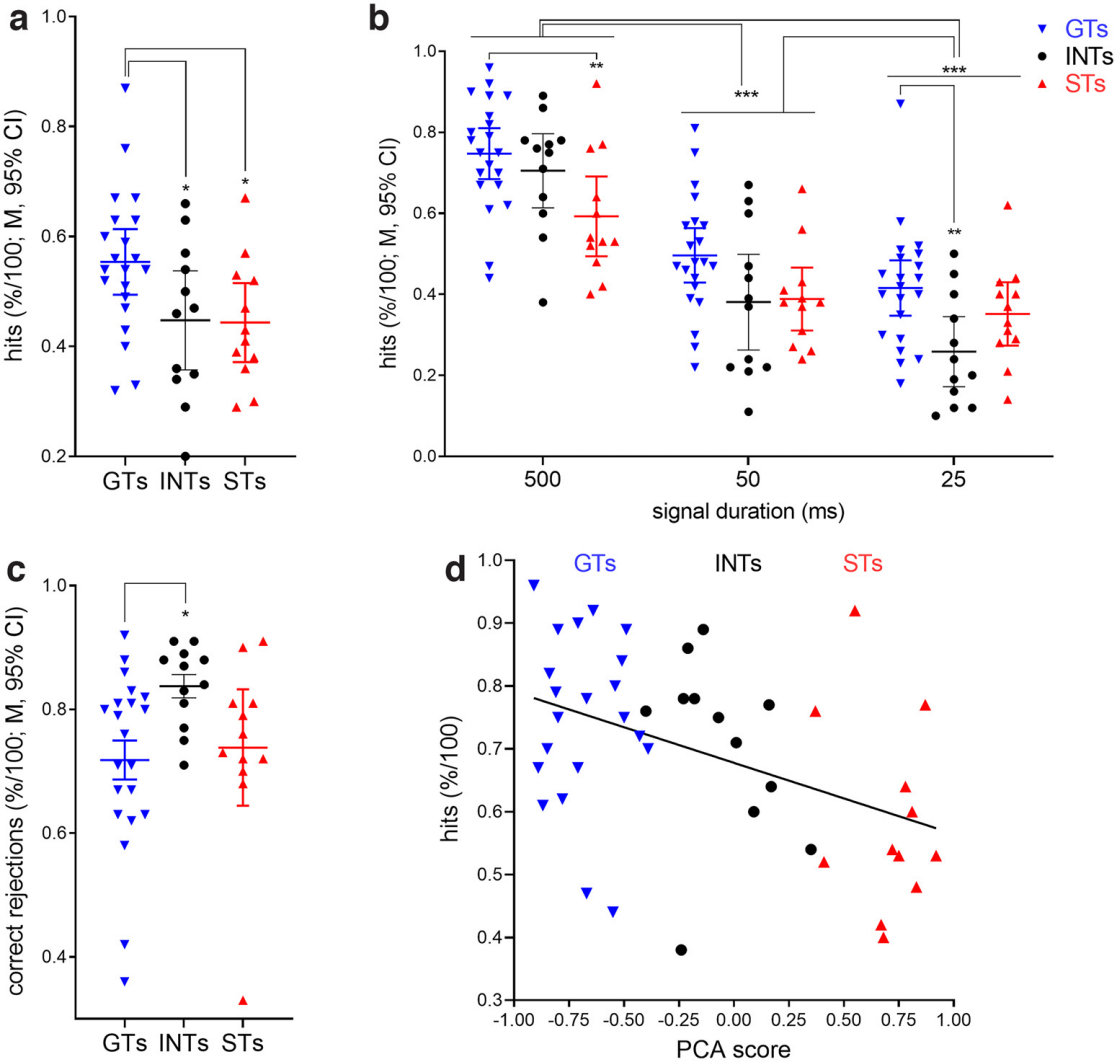
Attentional performance of GTs, INTs, and STs. Unless noted otherwise, these and subsequent data figures show individual values, means and 95% confidence intervals (CIs). Furthermore, figures depict the results of *post hoc* multiple comparisons (*p < 0.05, **p < 0.01, ***p < 0.001; see Results for ANOVAs, effect sizes, and other major statistical findings). ***a***, A main effect of phenotype on the relative number of hits reflected that GTs scored more hits than INTs and STs. ***b***, Across all phenotypes, fewer hits were scored to 50- and 25-ms signals when compared with longest signals. Moreover, STs had fewer hits to the longest signals when compared with GTs. ***c***, The relative number of correct rejections did not differ between GTs and STs but was unexpectedly higher in INT relative to GTs (see Results for a follow-up of this finding on response bias). ***d***, depicts the significant correlation between the relative number of hits to longest signals and PCA scores. More positive PCA scores (greater expression of sign-tracking behavior) predicted lower detection rates.

The analysis of the relative number of hits revealed main effects of phenotype and a significant interaction between the effects of phenotype and signal duration [main effect of phenotype: *F*_(2,42)_ = 3.91, *p *=* *0.03 (Fig. 2*a*); signal duration: *F*_(2,84)_ = 196.73, *p *<* *0.001; phenotype × duration: *F*_(4,84)_ = 4.84, *p *=* *0.001 (Fig. 2*b*)]. *Post hoc* multiple comparisons indicated that GTs scored significantly more hits than INTs (Cohen’s *d* = 0.77) and STs (*d*: 0.89; main effect illustrated in Fig. 2*a*), that all rats detected fewer 50-ms and 25-ms signals than 500-ms signals, and that STs scored fewer hits in response to the longest signals than GTs (Fig. 2*b*). Although male rats scored more hits than female rats (*F*_(1,39)_ = 8.01, *p *=* *0.007; M ± SD: males: 54.02 ± 13.08%; females: 42.58 ± 11.39%), the effect of phenotype on hits were not moderated by sex (all two-way and three-way interactions involving the factor sex, *p *>* *0.14).

A main effect of phenotype on the relative number of correct rejections (*F*_(2,44)_ = 3.47, *p *=* *0.04) reflected that INTs scored more correct rejections than GTs (see further below for more analysis of this finding). However, the relative number of correct rejections did not differ between STs and GTs (Fig. 2*c*). Omissions remained relatively low and neither differed between the phenotypes (*F*_(2,44)_ = 1.44, *p *=* *0.25), nor sexes (*F*_(1,44)_ = 0.01, *p *=* *0.96), nor did the two factors interact significantly (*F*_(2,44)_ = 0.45, *p *=* *0.64; grand M ± SD: 4.45 ± 4.50% omissions).

#### Hit rates correlated with PCA scores

Across all three phenotypes, the degree of sign tracking versus goal tracking (PCA score) predicted hit rates to longest signals (*R*^2^ = 0.20, *p *=* *0.002; Fig. 2*d*). For each increase of the PCA score by 0.25, indicating a shift away from a preference for approaching the magazine toward approaching and contacting the CS (that is, shifting toward more sign tracking), the hit rate decreased by ∼3%.

### Do INTs form a separate phenotype?

Previous studies have rarely determined the behavioral and neurobiological status of INTs. Overall, INTs scored fewer hits than GTs (Cohen’s *d* = 0.89; Fig. 2*a*), but this difference was not consistently apparent when hits were calculated separately by signal duration (Fig. 2*b*), and INTs did not score significantly more hits than STs. Furthermore, the correlation shown in Figure 2*e* required inclusion of the data from INTs to reach significance. Thus, the present data largely support the view that, in terms of their attentional performance, INTs fit in-between GTs and STs, as opposed to reflecting a separate phenotype characterized by a performance pattern distinct from the more extreme phenotypes.

However, INTs correctly rejected more nonsignal trials than GTs (Fig. 2*d*), suggesting the possibility that the performance of INTs was influenced by a relatively more conservative response bias than in GTs (that is, a relatively greater propensity for reporting the absence of signals). Therefore, we calculated a signal detection theory-derived measure of response bias [*B”_D_*; scores ranging from −1 (liberal bias) to +1 (conservative bias); see Materials and Methods]. This analysis indicated a significant effect of phenotype (*F*_(2,44)_ = 3.53, *p *=* *0.04), reflecting that GTs applied a significantly more liberal bias than STs (M ± SEM; GTs: 0.32 ± 0.10, STs: 0.68 ± 0.0; LSD: *p *=* *0.01), meaning that GTs exhibited a relatively greater propensity than STs to report the presence of a signal (validly or not). However, *B”_D_* scores of INTs did not differ from those of the other two phenotypes (INTs: 0.52 ± 0.11; both *p *>* *0.16). Together, these data support the view that, consistent with their intermediate PCA scores, the level of attentional performance of INTs was situated between the two, more extreme phenotypes.

### Attentional performance decrement in STs

Following selective lesions of the basal forebrain cholinergic system, the hit rates of rats were previously found to be greatly suppressed to ∼30% hits across signal durations (McGaughy et al., 1996). Compared with such a fundamental and persistent loss of the capacity for detecting signals, and albeit STs scored significantly fewer hits than GTs (above), the distribution of the present hit scores pointed to less profound, and perhaps more complex, cognitive-behavioral mechanisms which may have contributed to the performance difference between the two phenotypes.

To further explore the origin of the relatively lower hit rates in STs, we investigated the rats’ performance over time on task, by extracting hit rates over 3 min trial blocks (yielding a total of 13 blocks per session). Inspection of the data from individual GTs and STs suggested that, in STs, hits to longest signals steeply declined toward the end of the session. On contrast, GTs tended to exhibit more stable levels of hit rates, with little or no decline toward the end of the session.

To quantify and test this possibility, we counted the number of 3-min blocks in which the individual rat scored above chance levels of hits, defined by >60% hits (>59 hits/100 trials at *p* = 0.5; *p < *0.044; binomial test). This count was obtained separately for the last five 3-min blocks of performance (blocks #9–13) and, as a control, for the five blocks preceding these last five (blocks #4–8).

**Figure 3.**
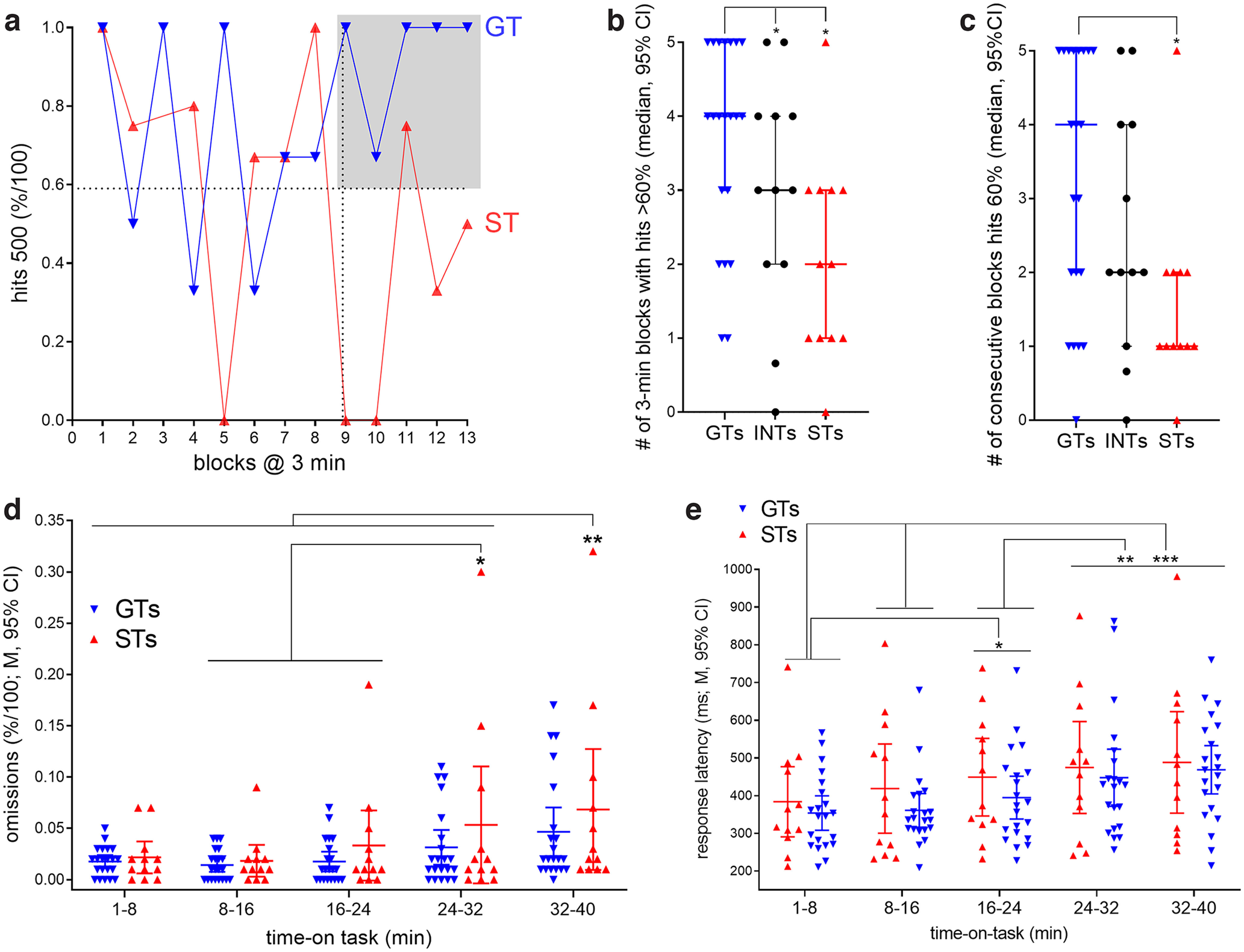
Decline in hits in STs toward the end of the session. Performance data were extracted from individual sessions and collapsed over 5-min blocks, yielding 13 blocks of trials. ***a***, Illustration of the relative number of hits to longest signals by a representative GT and a ST. In contrast to the GT, the ST’s hit rate was less stable, fluctuating between high (>60%) and low levels (<30%) particularly during the last five blocks of trials. To quantify this observation, for the last five blocks we counted the number of blocks with above-chance hit rates (<60%; gray area in ***a***) and the number of consecutive blocks with >60% hits separately for STs and GTs (shown in ***b*** and ***c***, respectively). The analysis of both measures indicated main effects of phenotype; multiple comparisons (Mann–Whitney *U* test; see Results) confirmed that in comparison to GTs, the hit rates of STs relatively rarely reached above-chance level (***b***), and if, rarely stayed at this level for longer than one block (***c***). ***d***, To assess a possible contribution of loss of motivation in STs to their decrease in hits, we also analyzed the relative number of trials during which rats did not press a lever (omissions; ***d***). Omissions overall remained relatively rare (less than ∼10% of trials), slightly but significantly increased toward the end of the session (see Results from *post hoc* comparisons in ***d***), but omissions did not differ between the phenotypes (no main effect and no interaction between the effects of block and phenotype). Likewise, the analysis of response latencies for hits to longest signals (time between extension of the lever and a lever press) indicated that response times increased for all animals toward later blocks (***e***), but that phenotype did not influence response latencies (no main effect and no interaction between the effects of block and phenotype).

Figure 3*a* depicts the hit rates of an individual GT and ST. Across the last five blocks of trials, the GT scored >60% hits in all blocks, while the ST had only one such block. Across all GTs and STs, we found main effects of phenotype on the number of blocks with above-chance hit rates (H(2) = 8.12, *p *=* *0.02 Kruskal–Wallis test; Fig. 3*b*), and on the number of consecutive blocks with above-chance hit rates (H(2) = 6.68, *p *=* *0.04; Fig. 3*c*). These main effects indicated significantly lower counts for STs and each of the two measures when compared with GTs. Moreover, multiple comparisons also indicated a lower number of blocks with above-chance hit rats in INTs relative to GTs (Fig. 3*b*). A similar analysis of hit rates from the preceding five blocks of trials (Fig. 3*a*, blocks #4–8) did not reveal such effects of phenotype (both *p *>* *0.31; data not shown). These findings indicated that compared with GTs, STs exhibited a robust decrement in performance toward the end of the test sessions, characterized by overall lower performance and rapid fluctuations between above-chance and below-chance performance. These results on time-on-task-associated decline in hit rates further confirmed the view that the performance of INTs can be seen as situated in-between GTs and STs, as opposed to forming a distinct attentional phenotype.

Although the number of errors of omission increased for all rats toward the end of the session (main effect of block: *F*_(4,124)_ = 8.55, *p *=* *0.001; Fig. 3*d*), importantly, the absence of a main effect of phenotype and of an interaction between the effects of block and phenotype (phenotype: *F*_(1,31)_ = 1.03, *p* = 0.32; phenotype × block: *F*_(4,124)_ = 0.58, *p *=* *0.68) did not suggest that a declining motivation to perform contributed to the decrement in hit rates in STs. To further assess a possible loss of motivation or other overt behavioral mechanisms which may have contributed to the late decrease in detection rates in STs, we also analyzed response times for hits to longest signals (time from extension of the lever to a lever press). This analysis rejected the possibility that one phenotype was slower than the other, in general or during individual blocks (main effect of phenotype: *F*_(1,31)_ = 0.75, *p *=* *0.39; block × phenotype: *F*_(4,124)_ = 0.23, *p *=* *0.92). All rats, regardless of phenotype, responded more slowly during later blocks when compared with earlier blocks (main effect of block: *F*_(4,124)_ = 6.78, *p *<* *0.001; for multiple comparisons, see Fig. 3*e*).

### Basal forebrain inhibition converts the attentional performance of GTs to that of STs

Because the level of attentional performance of INTs was situated between STs and GTs, as opposed to indicating a separate phenotype, we restricted the determination of effects of CNO to the two extreme phenotypes. Furthermore, as neither our prior experiments (Kucinski et al., 2019; Avila et al., 2020), nor the experiments by other investigators (Tran et al., 2020; Norman et al., 2021) indicated the presence of off-target effects of CNO in rodents transfected with a control virus or in nontransfected rodents, such a control was not included here.

**Figure 4.**
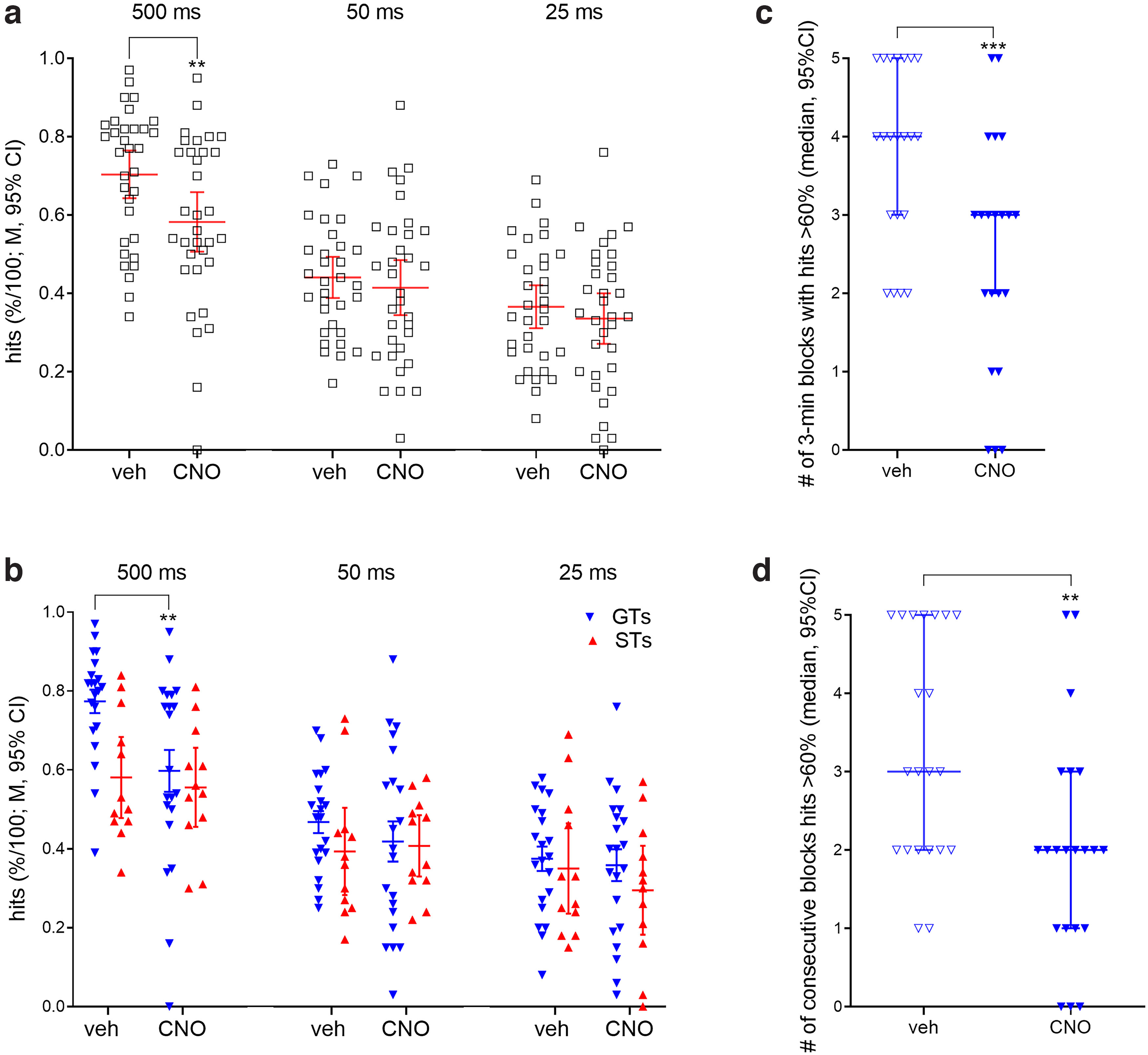
CNO administration reduced hit rates in GTs, but not STs, and specifically during the last 15 min of trials of the test session. ***a***, A significant interaction between the effects of CNO and signal duration reflected that CNO suppressed hit rates to longest, but not 50- and 25-ms signals. Hits to the shorter signals approached chancel level, thereby limiting the demonstration of effects of CNO. A three-way interaction (phenotype × CNO × signal duration) originated from a selective effect of CNO on the hits to longest signals in GTs (***b***). The CNO-induced loss of hits to longest signals manifested specifically during the last 15 min of performance, as indicated by a significant reduction of the number of 3-min blocks with >60% hits (***c***) and of consecutive blocks with above-chance hit rates (***d***; Wilcoxon test, see Results). A similar analysis of the number of blocks with >60% hits, and the number of such consecutive blocks, from the preceding 15 min of trials (blocks 4–8) did not indicate significant effects of CNO (data not shown).

The effects of CNO significantly interacted with effects of signal duration (*F*_(2,62)_ = 3.34, *p *=* *0.04), reflecting that CNO suppressed the hit rates to longest, but not 50-ms and 25-ms signals (Fig. 4*a*). Furthermore, a three-way interaction (phenotype × drug × signal duration: *F*_(2,62)_ = 3.64; *p *=* *0.03) suggested that the efficacy of DREADD activation differed between the phenotypes and hits to the three signal durations. Follow-up two-way ANOVAs on the effects of drug and signal duration for the individual phenotype indicated that DREADD activation reduced hit rates in GTs but not STs (GTs: *F*_(1,20)_ = 7.32, *p *=* *0.01; STs: *F*_(1,11)_ = 0.21, *p *=* *0.66). *Post hoc* multiple comparisons isolated the effects of CNO to hits to 500-ms signals (*t*_(20)_ = 18.74; *p *=* *0.01; Cohen’s *d* = 0.90; Fig. 4*b*). Administration of CNO did not affect the relative number of correct rejections (*F*_(1,31)_ = 0.81, *p *=* *0.38) but increased the relative number of omissions in all rats (main effect: *F*_(1,31)_ = 5.64, *p*= 0.02; vehicle: 4.06 ± 0.85% omitted trials; CNO: 11.52 ± 3.11%).

Above, we demonstrated that the reduced hit rate in STs, when compared with GTs, manifested specifically during the last five 3-min blocks of trials, suggesting a profound performance decrement across time-on-task. We therefore determined whether, in GTs, CNO administration similarly suppressed hit rates to 500-ms signals specifically during the last 15 min of the test session. As shown in Figure 4*c*, CNO administration to GTs significantly reduced the number of 3-min blocks with hit rates >60% (Wilcoxon test; *Z *=* *3.31, *p *=* *0.0009). Furthermore, CNO also reduced the number of consecutive blocks with hit rates >60% (*Z *=* *3.07, *p *=* *0.002; Fig. 4*d*). Neither measure was significantly reduced by CNO during the five 3-min blocks that preceded the last 15 min of performance (blocks #4–8; both *Z *<* *1.87, both *p *>* *0.06; data not shown). Thus, suppressing the activity of the basal forebrain in GTs yielded a decline in attentional performance specifically during the last 15 min of trials, mirroring the performance of naive STs during this period.

#### Viral transfection efficacy in the HDB predicted the behavioral efficacy of CNO in GTs

**Figure 5.**
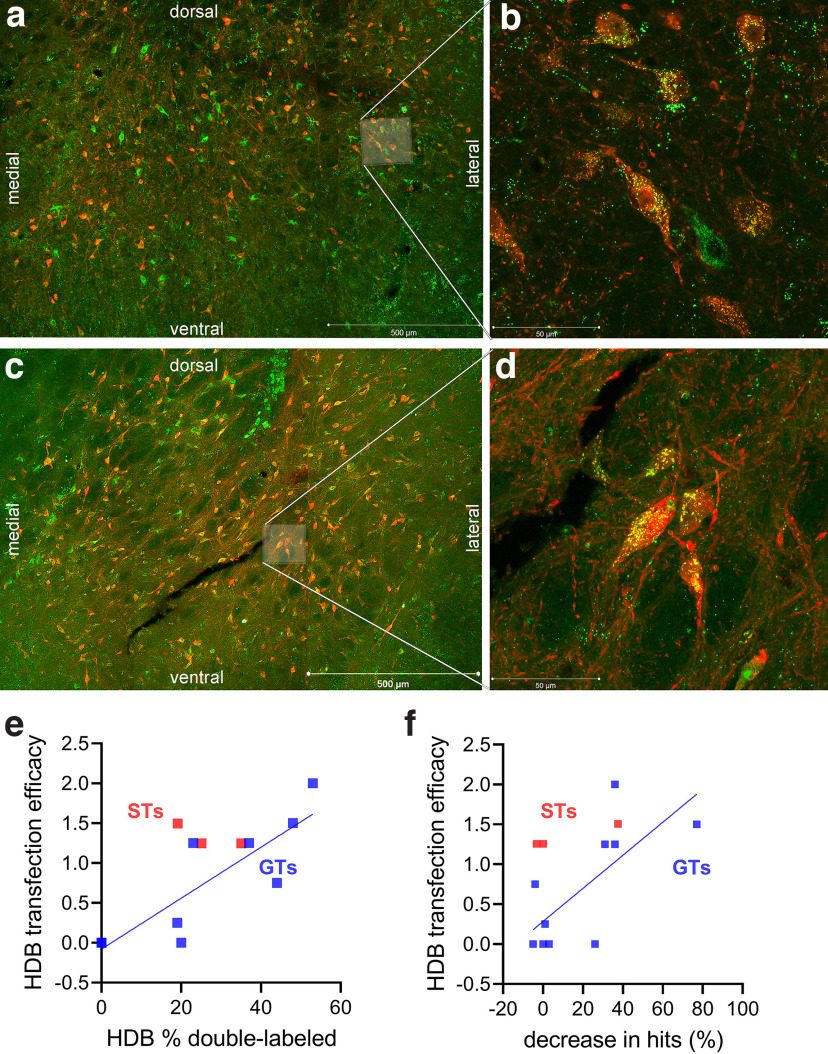
Visualization of DREADD expression in HDB cholinergic and noncholinergic neurons (m-cherry, appearing red, indicates the expression of the DREADD construct; Alexa 488, appearing green, indicates the presence of the VAChT). ***a***, ***c***, Low-magnification photomicrographs (500-μm scale inserted) of a coronal section showing the HDB of a GT and a ST, respectively. These photomicrographs exemplify the dense presence of magnocellular cholinergic neurons (green) as well as numerous neurons expressing the DREADD construct (red) or both markers. The areas marked by a whitish overlay in ***a*** and ***c*** are magnified in ***b*** and ***d***, respectively, and show several double-labeled neurons (yellow puncta). In ***b***, a cholinergic neuron (green) not expressing the DREADD-reporter is also present. For the 10 GTs selected for histologic analyses, viral transfection efficacy and the degree of double-labeling was significantly correlated for counts from the HDB (***e***) but not for the other two subregions. Moreover, the efficacy of DREADD activation, in terms of decreases in hits, was significantly correlated with viral transfection efficacy in the HDB (***f***) but not for counts obtained from the other two subregions. In none of the three subregions did the proportion of cholinergic neurons also expressing the DREADD correlate with decreases in hits (data not shown). ***e*** and ***f*** also depict the transfection efficacy and decreases in hits in the three STs selected for histologic analyses based on transfection efficacies that were comparable to those in GTs which showed the greatest effects of DREADD activation on SAT performance.

As detailed in Methods, DREADD-positive cells (total number and proportion of cholinergic neurons) were counted in three major regions of the basal forebrain projecting to cortical regions (nbM, SI, HDB). For this analysis we selected the brains of two subgroups of GTs, five each, which exhibited the largest and smallest, respectively, decreases in the relative number of hits following the administration of CNO (median percent decrease of the 5 rats with the largest decreases in hits: 43.55%; smallest: 5.01%). The total number of DREADD-positive cells per subregion was categorized into five classes of transfection efficacy. Furthermore, the percent of neurons co-localizing m-Cherry (DREADD expression reporter) and Alexa 488 (VAChT-positive) was calculated for each of the three subregions of the basal forebrain. Figure 5*a*,*b* shows a low-magnification microphotograph of the HDB of a GT and a ST, respectively, exemplifying the relative dense packing of cholinergic neurons in this area and the widespread expression of the DREADD construct in cholinergic and noncholinergic neurons. The magnified areas shown in Figure 5*b*,*d*, respectively, show several cholinergic cells expressing the DREADD construct. The overall transfection efficacy and the percent double-labeled neurons were significantly correlated for counts taken from the HDB of GTs (ρ = 0.91, *p *=* *0.0009; Fig. 5*e*), but not for the other two subregions (both *p *>* *0.22). Furthermore, correlations between the CNO-induced decreases in hit rates of GTs and transfection efficacy reached significance solely for counts taken from, and averaged across the hemispheres, the HDB (ρ = 0.73, *p *=* *0.021; Fig. 5*f*; SI, nbM: both *p *>* *0.14). However, the percent of cholinergic neurons that also expressed the DREADD significantly correlated with decreases in hits neither for counts taken from the HDB (ρ = 0.57, *p *=* *0.08) nor for the other two subregions (both *p *>* *0.12).

To test the possibility that CNO remained relatively ineffective in STs because of a potential group-wide failure to produce DREADD transfection efficacies that were comparable to those seen in GTs, we selected three STs with HDB transfection efficacy scores that were within the range of the five GTs that showed the largest CNO-induced decreases in hits (Fig. 5*c*,*d* for an illustration of such a case; see also Fig. 5*e*,*f*, red symbols). CNO administration shifted these animals’ hit rates (differences to vehicle) by −3.22%, +0.19%, and −37.45% respectively (the latter decrease in hits was the largest effect of CNO seen in STs; median difference in STs: +2.83%). Thus, DREADD expression efficacy in the HDB predicted the behavioral efficacy of DREADD activation GTs, but not STs.

## Discussion

Male and female rats were screened using the PCA test to assign the three phenotypes (STs, INTs, and GTs). The rats of these three phenotypes acquired the SAT at statistically similar rates. However, once asymptotic performance was achieved, STs scored fewer hits than GTs while rejecting nonsignals at the same rate as GTs. Furthermore, the relatively fewer hits by STs originated exclusively from the last 15 min of a ∼40-min-long performance session. Importantly, the mounting instability of the detection rate of STs toward the end of the session was not associated with an increase in errors of omission. Hit rates across the phenotypes were significantly correlated with PCA scores (the more sign tracking, the fewer hits). In general, INTs do not seem to form a discrete attentional phenotype; rather, their performance was consistent with the PCA-derived classification as intermediates. In GTs, chemogenetic inhibition of the basal forebrain, specifically of the HDB, reproduced the late-session instability of the detection rate seen in untreated STs. DREADD activation remained statistically ineffective in STs. The total number of HDB neurons transfected with the DREADD construct, but not the number of cholinergic neurons positive for the DREADD-reporting fluorophore, correlated with the effects of DREADD activation on hits. The discussion below focuses on the nature of the attentional phenotypes of STs and GTs, on the collective functions of HDB cholinergic and noncholinergic projections to cortex in attention, and on the potential impact specifically of cholinergic dysregulation in STs on these functions.

Because the reduction of hit rats following inhibition of the basal forebrain was limited to longest signals in GTs, the role of variable signal intensity in the construction of sustained attention tasks requires comment. To effectively tax the capacity for sustained attention, signals of variable intensity serve to limit the manifestation of a fixed perceptual sensitivity threshold and, thereby, increase mental workload and foster a performance decline over time (Parasuraman and Mouloua, 1987; Parasuraman et al., 1987; Temple et al., 2000; Berardi et al., 2001). An effective dynamic signal intensity range typically would include low-intensity signals that yield hit rates approaching chance level. Thus, different hit rates to individual signal intensities per se do not indicate separate attention modes; rather, they reflect the impact of variations of one task parameter employed to tax sustained attention performance (see also Lim et al., 2010; Chebolu et al., 2022). Variations of event rate, the insertion of nonsignal trials, and rewarding not just hits but also correctly reported absences of signals (correct rejections) are additional means to tax such performance (McGaughy and Sarter, 1995; Echevarria et al., 2005).

In contrast to the on-off visual signals used in the SAT, the perception of more complex visual stimuli such as orientation-specific gratings or stimuli requiring the detection of incremental contrast changes or binocular processing, critically depend on the integrity of primary visual cortical regions (Kang et al., 2014; Sheynin et al., 2020; Goldbach et al., 2021). Thus, it seems unlikely that inhibition of basal forebrain inputs to primary visual regions (Huppé-Gourgues et al., 2018), and thus primarily perceptual effects, contributed to the decrease in hits in GTs. Furthermore, such potential, primary perceptual effects of DREADD inhibition would have been expected to manifest throughout a SAT session and not just exclusively toward the end of such sessions, and it would have been expected to likewise manifest in STs. As will be discussed below, decreases in detection performance that depend on the time-on-task are hypothesized to reflect effects on attentional control mechanisms, mediated in part via inhibition of basal forebrain inputs to frontal-parietal cortical regions (Serences et al., 2005; Rossi et al., 2007).

STs were previously demonstrated to attend to external cues in accordance with a bias for bottom-up, or cue salience-driven, processing. In contrast, GTs may have minimized the influence of cue salience on hit rates, perhaps by applying relatively more goal-directed, decisional biases (Phillips and Sarter, 2020). We may also exclude a relatively lower motivation to perform as a source of the end-of-session decline in STs, as omissions remained low overall and did not differ between the phenotypes. We hypothesize, therefore, that the performance difference between STs and GTs toward the end of test sessions reflects, in STs, an attention decrement that was contrasted with a relatively superior and stable control of attentional performance in GTs.

There is a rich, albeit largely descriptive and speculative literature on the cognitive capacities required to sustain attention over long periods of time, and on the mechanisms responsible for time-on-task associated increases in distractibility, impulsive action, the rate of attentional lapses or, to employ another theoretical construct, attentional fatigue. Furthermore, time-on-task-dependent decrements in attentional performance have often been attributed to weak top-down control, that is, to the decline of a set of operations that include: maintaining task rules and goals in working memory; error monitoring; weighing reward and reward loss against levels of motivation; enhanced cue processing and distractor filtering; and suppression of competitive behaviors (Parasuraman and Mouloua, 1987; Hopfinger et al., 2000; Miller and Cohen, 2001; Miller and D’Esposito, 2005; Baluch and Itti, 2011; Head and Helton, 2014; Esterman and Rothlein, 2019). Theories which attempted to break out of the circular logic that attributes vigilance decrements to weak top-down control, and solid top-down control to the relative superiority in some mental capacity, have remained rare and typically have continued to pose limited capacities for deploying sustained attentional effort, specifically when computing the benefits of alternative action (Braver, 2012; Kurzban et al., 2013; Thomson et al., 2015; Esterman and Rothlein, 2019; Chebolu et al., 2022). Thus, the time-on-task associated decline of performance of STs, on the one hand, can readily be discussed in terms of relatively poor top-down control (or, conversely, of the relatively strong top-down control by GTs) but, on the other hand, remains mechanistically unsettled (for a computational model on the costs of maintaining elevated attention across multiple trials of the SAT see Chebolu et al., 2022).

The evidence in support of a cholinergic role in the detection of signals and of a cholinergic abnormality of STs may provide a more conclusive foundation for understanding the time-on-task decrement seen in STs. Early studies on the effects of basal forebrain cholinergic lesions on hit rates (McGaughy et al., 1996), trial-specific recordings of cholinergic activity and associated oscillatory activity (Parikh et al., 2007; Howe et al., 2013, 2017) and, more recently, effects of optogenetic generation or suppression of cholinergic activity on hit rates (Gritton et al., 2016), have consistently indicated that scoring hits in such tasks, but not correctly reporting the absence of signals, requires cholinergic activation of frontal cortices. Collectively, this evidence has given rise to the view that the release of acetylcholine (ACh) in such tasks is bound, on the scale of seconds, to specific trials (hits), as opposed to prior views that ACh primarily modulates target regions, over minutes, to influence a behavioral state (for more discussion see Sarter and Lustig, 2020). In such tasks, cholinergic signaling evokes high-frequency oscillations via stimulation of muscarinic M1 postsynaptic receptors, and this effect is thought to mobilize neuronal networks involved in cue-based decisions to execute a hit (Howe et al., 2017).

Over longer periods of time, cholinergic synapses in STs may exhibit a reduction in signaling capacity, for the following reasons. Studies addressing a potential deficiency of the capacity for cholinergic signaling in STs demonstrated that, at baseline, the plasma membrane density of CHTs is similar in STs and in GTs (Koshy Cherian et al., 2017). However, depolarization of synaptic terminals in cortex *in vivo*, over 15 min, tripled ACh release in GTs but only doubled it in STs, and this attenuated release in STs was attributed to a reduced rate with which CHTs translocate from intracellular domains into the synaptosomal plasma membrane (Koshy Cherian et al., 2017). This evidence may be consistent with the present finding that, during the early trials of SAT sessions, hit rates did not differ between STs and GTs. In contrast, following an extended period of continuous performance, approaching 120 trials total, the CHT trafficking deficit in STs may have limited the capacity of cholinergic synapses to continue supporting the scoring of hits. Thus, this neuro-mechanistic interpretation attributes the performance decrement in STs to a progressively declining capacity of cholinergic neurons to support the detection of signals (see further below for a discussion of interactions between cholinergic and noncholinergic basal forebrain systems).

The proposed neuro-mechanistic foundation of the attentional control decline seen in STs suggests significant limitations for conventional strategies designed to rescue or improve the SAT performance of STs. Stimulation of cholinergic neurons may not overcome the fundamental capacity limit imposed by the CHT outward trafficking limit. Likewise, conventional strategies to elevate synaptic and extra-synaptic levels of ACh by, for example, inhibiting acetylcholinesterase, would disregard the highly phasic nature of cholinergic communication (Sarter and Lustig, 2020). Elevated levels of extracellular ACh would further disrupt the synaptic dialogue via stimulation of autoinhibitory presynaptic muscarinic M2 receptors (Sarter, 2015). More promising strategies to effectively enhance cholinergic processing and rescue the SAT performance of STs may require first a clarification of the cellular basis of the CHT trafficking deficit in STs, and a normalization of the activity-dependent population of synaptic plasma membrane with functional CHTs.

The present histologic analyses indicated that inhibition of the HDB as a whole, not just of its cholinergic projections to medial frontal and retrosplenial cortices (Gyengesi et al., 2013; Gielow and Zaborszky, 2017), was closely associated with the CNO-induced decrease in hit rates in GTs that manifested toward the end of the test sessions. This finding may be considered to conflict with the focus on cholinergic signaling capacity limits as a key neuronal mechanism underlying the relatively poor hit rates in STs late in the session. However, the impact of neither a CHT abnormality, nor the effects of selective optogenetic inhibition of basal forebrain cholinergic neurons (Gritton et al., 2016) or of chemogenetic inhibition of selectively these neurons, are likely to remain restricted to cholinergic activity (Miesenbock, 2009). Indeed, local interconnectivity within the basal forebrain between cholinergic and noncholinergic cells, in part via multiple populations of interneurons, as well as long-loop feedback connections directly from cortex and via multiple intermediate regions, together are extremely likely to co-modulate the activity of cholinergic and noncholinergic projections (Duque et al., 2000; Sarter and Bruno, 2002; Zant et al., 2016; Gielow and Zaborszky, 2017; Yang et al., 2017; Anaclet et al., 2018). Consistent with this view, results from numerous experiments indicated that cholinergic and noncholinergic projection systems function in overlapping or complementary ways to support complex behaviors, including SAT performance (Burk and Sarter, 2001; Lin et al., 2006; Lin and Nicolelis, 2008; Avila and Lin, 2014; Tingley et al., 2018; Hwang et al., 2019).

CNO-induced DREADD activation in GTs reproduced the time-on-task associated decrease in hit rates seen in untreated STs. Although the relative subtlety of this effect may have been unexpected (but see Miesenbock, 2009), the performance of GTs may have been partially protected against the effects of DREADD activation based on compensatory top-down capacities. Eventually, late into the test session, this “cognitive reserve” may have remained insufficient to stem the impact of inhibition of the basal forebrain, including inadequate reloading of cholinergic synapses (above), yielding a decline in detection rates. CNO did not further reduce the hit rate in STs (Fig. 4*b*), despite equivalent transfection efficacy in the HDB (see Results). Previous studies reported the lack of increases in ACh release in STs in response to exposure to a Pavlovian cocaine cue (Pitchers et al., 2017b), the absence of effects of basal forebrain cholinergic lesions on the response of STs to a cocaine reinstatement cue (Pitchers et al., 2017a), and of effects of basal forebrain chemogenetic inhibition on complex movement control in STs (Kucinski et al., 2019). The results from these prior and the present study collectively suggest that STs can perform these tasks, at least for a relatively limited time and to a degree, without depending essentially on recruiting functionally acting top-down, basal forebrain-cortical and cortical-subcortical systems (Campus et al., 2019). The finding that humans expressing a genetic CHT variant, known to limit cholinergic signaling under taxing conditions (Donovan et al., 2022), perform the SAT at wild-type levels but, in contrast to the latter, without activating a key cortical node of the brain’s dorsal attention system (Berry et al., 2015) is consistent with the idea that variations in cholinergic capacities are associated with differential cognitive strategies and resources deployed to sustain attention.

The present results extend the conceptualization of sign tracking and goal tracking as behavioral indices of broader opponent traits, or cognitive styles (Sarter and Phillips, 2018). Limitations in the ability to sustain attention, including the contributions of attentional lapses and attentional fatigue to performance decline, are considered essential components in the relative weakness of STs to resist approaching and operating drug cues, and of the cognitive vulnerabilities of humans with substance use disorder (Tomasi et al., 2007; Field and Cox, 2008; Romens et al., 2011). Moreover, STs exhibit a bias for the (dopaminergic) processing of the motivational attributes of drug cues (for evidence from human sign-trackers see Colaizzi et al., 2020; Schad et al., 2020; Cope et al., 2023), thereby further increasing the perceived salience of such cues and reducing resistance toward approaching and using such cues. In contrast, GTs deploy more effective top-down strategies to sustain attention and, more generally, seem to employ a “colder” analysis of the utility of cues for goal-directed behavior, combined with relatively low levels of (dopaminergic) processing of incentive values of drug cues (Pitchers et al., 2017b). In addition to the relevance of these animal models of opponent cognitive styles to study the neuronal mechanisms underlying vulnerability for and resistance against, respectively, repeated addictive drug use, research on STs and GTs may assist in revealing differential risks for a wide range of neuropsychiatric disorders (Robbins et al., 2012).

## Synthesis

Reviewing Editor: Erica Glasper, The Ohio State University Decisions are customarily a result of the Reviewing Editor and the peer reviewers coming together and discussing their recommendations until a consensus is reached. When revisions are invited, a fact-based synthesis statement explaining their decision and outlining what is needed to prepare a revision will be listed below. The following reviewer(s) agreed to reveal their identity: Gregor Rainer.

<b>Synthesis</b>: In this manuscript, attempt to determine how chemogenetic inhibition of the basal forebrain alters attention in goal- and sign-trackers. Goal- versus sign-trackers is a popular mode because it examines individual differences in learning during autoshaping. Overall, the authors suggest that goal-trackers have increased attention during the task, but this is impaired by chemogenetic inhibition of the basal forebrain system. The reviewers find the topic of great interest; however, the current version of the manuscript is difficult to read. A major restructuring of the manuscript, addressing the points below, may help increase readability and understanding.

Major Points

1. Please consider rewording the title, as the authors did not demonstrate that they converted goal-trackers into sign-trackers.

2. The design and possible validity of the study is questioned because of the lack of DREADD effects on the 25 and 50ms conditions. While the authors suggest this may be due to performance at chance levels under these conditions, this is cause for concern. Specifically, the design of this attention task was to demonstrate that rats really do not need to attend unless the duration of the light was very short, however, all the observed differences were only present for 500 ms. This is concerning and may suggest that differences may not be related to attention.

3. There is concern that the goal-trackers and sign-trackers may perform differently on the attention task for reasons other than those related to differences in attention. The authors should discuss the possibility that the goal-trackers may remain at the food cup which could increase the likelihood that they are see the light and get more “hits”. In the same light, sign-trackers may remain oriented to the levers, which may limit their ability to see the light as well as the goal trackers. The authors should consider how locomotion may different between goal- and sign trackers. Does one switch back and forth between where the lever comes out and where food is delivered more than the other?

4. In addition to the authors current hypothesis that goal trackers pay more attention, the authors should also consider the hypothesis that sign-trackers have a stronger bottom-up attention to sensory stimuli.

5. The authors should improve documentation of the histological correlations, particularly when it comes to the sign-tracking group. The authors should further address why the sign-tracking rats lost motivation during the last trial blocks.

6. The authors should address why most of the observed effects are dependent on examining trials at the end of the session. Again, this may suggest that motivation, rather than attention, is driving the observed outcomes. While the authors state that motivation is not involved because the number of omissions is not different, however, poorer performance can also reflect changes in motivation.

7. 3-way ANOVAs are difficult to interpret and rely on several assumptions. The authors should determine whether 3-way ANOVAs are necessary to best represent the main findings.

Minor Points

<i>Overall</i>

1. The authors report hit rates; however, it may be desirable to compute d– since it considers aggregate performance and not hit rate alone. Interesting relationships between sign- and goal-tracking groups are missed by lumping latency, response bias, and probability into one measure. Individual analysis should be considered.

2. Please report reaction times between sign- and goal-trackers. It is possible that fast impulsive choices might lead to poorer decisions that hits compared to misses. When reporting reaction times, please determine if reaction time changed over the course of the session (i.e., did reaction times slow as the sessions went on?).

3. The authors should consider adding a schematic of the testing apparatus and include CS, magazine, and lever placement would be useful.

4. Please include a description of how PCA was computed.

5. The authors should include discussion of the work from the Elvire Vaucher lab, as their work relates to cholinergic basal forebrain effects on vision.

6. Please define all abbreviations, even within the abstract.

7. The introduction could be improved by adding descriptions of sign- and goal-tracking. This will help unfamiliar readers increase understanding.

<i>Specific</i>

1. Line 67 - Please clarify whether activation of the cholinergic system to rescue attentional control would be an option. Also, discuss whether performance is already compromised in goal-trackers and if a rescue with an appropriate intervention would be important to note.

2. Lines 362-369 - It is unclear why the authors would suggest that differences between goal-trackers and sign-trackers would not be related to sex when the current data would suggest so. Please discuss why the present findings were discounted based on the work of Pitchers et al., 2015.

3. Line 395 - Please operationally define “omission”. Does this equal a “miss”?

4. Lines 418-422 - Please describe what it means for the goal-trackers to demonstrate a “more liberal bias”. As currently written, this section is difficult to interpret as it relates to “phenotypes).

5. Line 424-426 - The author should clarify what is meant by “removal of the basal forebrain cholinergic system”. Was the system removed of deactivated? Were specific cell types targeted? Also, please clarify whether “hit rates” or signal detection rates” were assessed.

## References

[B1] Agostinelli LJ, Geerling JC, Scammell TE (2019) Basal forebrain subcortical projections. Brain Struct Funct 224:1097–1117. 10.1007/s00429-018-01820-6 30612231 PMC6500474

[B2] Anaclet C, De Luca R, Venner A, Malyshevskaya O, Lazarus M, Arrigoni E, Fuller PM (2018) Genetic activation, inactivation, and deletion reveal a limited and nuanced role for somatostatin-containing basal forebrain neurons in behavioral state control. J Neurosci 38:5168–5181. 10.1523/JNEUROSCI.2955-17.2018 29735555 PMC5977448

[B3] Armbruster BN, Li X, Pausch MH, Herlitze S, Roth BL (2007) Evolving the lock to fit the key to create a family of G protein-coupled receptors potently activated by an inert ligand. Proc Natl Acad Sci U S A 104:5163–5168. 10.1073/pnas.0700293104 17360345 PMC1829280

[B4] Arvidsson U, Riedl M, Elde R, Meister B (1997) Vesicular acetylcholine transporter (VAChT) protein: a novel and unique marker for cholinergic neurons in the central and peripheral nervous systems. J Comp Neurol 378:454–467. 10.1002/(SICI)1096-9861(19970224)378:4<454::AID-CNE2>3.0.CO;2-1 9034903

[B5] Avila C, Kucinski A, Sarter M (2020) Complex movement control in a rat model of Parkinsonian falls: bidirectional control by striatal cholinergic interneurons. J Neurosci 40:6049–6067. 10.1523/JNEUROSCI.0220-20.2020 32554512 PMC7392507

[B6] Avila I, Lin SC (2014) Motivational salience signal in the basal forebrain is coupled with faster and more precise decision speed. PLoS Biol 12:e1001811. 10.1371/journal.pbio.1001811 24642480 PMC3958335

[B7] Ballinger EC, Ananth M, Talmage DA, Role LW (2016) Basal forebrain cholinergic circuits and signaling in cognition and cognitive decline. Neuron 91:1199–1218. 10.1016/j.neuron.2016.09.006 27657448 PMC5036520

[B8] Baluch F, Itti L (2011) Mechanisms of top-down attention. Trends Neurosci 34:210–224. 10.1016/j.tins.2011.02.003 21439656

[B9] Berardi A, Parasuraman R, Haxby JV (2001) Overall vigilance and sustained attention decrements in healthy aging. Exp Aging Res 27:19–39. 10.1080/036107301750046124 11205528

[B10] Berry AS, Demeter E, Sabhapathy S, English BA, Blakely RD, Sarter M, Lustig C (2014) Disposed to distraction: genetic variation in the cholinergic system influences distractibility but not time-on-task effects. J Cogn Neurosci 26:1981–1991. 10.1162/jocn_a_00607 24666128 PMC4445375

[B11] Berry AS, Blakely RD, Sarter M, Lustig C (2015) Cholinergic capacity mediates prefrontal engagement during challenges to attention: evidence from imaging genetics. Neuroimage 108:386–395. 10.1016/j.neuroimage.2014.12.036 25536497 PMC4469545

[B12] Berry AS, Sarter M, Lustig C (2017) Distinct frontoparietal networks underlying attentional effort and cognitive control. J Cogn Neurosci 29:1212–1225. 10.1162/jocn_a_01112 28253080 PMC5920788

[B13] Black YD, Maclaren FR, Naydenov AV, Carlezon WA, Baxter MG, Konradi C (2006) Altered attention and prefrontal cortex gene expression in rats after binge-like exposure to cocaine during adolescence. J Neurosci 26:9656–9665. 10.1523/JNEUROSCI.2391-06.2006 16988036 PMC4203339

[B14] Braver TS (2012) The variable nature of cognitive control: a dual mechanisms framework. Trends Cogn Sci 16:106–113. 10.1016/j.tics.2011.12.010 22245618 PMC3289517

[B15] Burk JA, Sarter M (2001) Dissociation between the attentional functions mediated via basal forebrain cholinergic and GABAergic neurons. Neuroscience 105:899–909. 10.1016/s0306-4522(01)00233-0 11530228

[B16] Campus P, Covelo IR, Kim Y, Parsegian A, Kuhn BN, Lopez SA, Neumaier JF, Ferguson SM, Solberg Woods LC, Sarter M, Flagel SB (2019) The paraventricular thalamus is a critical mediator of top-down control of cue-motivated behavior in rats. Elife 8:e49041. 10.7554/eLife.49041 31502538 PMC6739869

[B17] Chebolu S, Dayan P, Lloyd K (2022) Vigilance, arousal, and acetylcholine: optimal control of attention in a simple detection task. PLoS Comput Biol 18:e1010642. 10.1371/journal.pcbi.1010642 36315594 PMC9648841

[B18] Cohen J (1988) Statistical power analysis for the behavioral sciences, Ed 2. Hillsdale: L. Erlbaum Associates.

[B19] Colaizzi JM, Flagel SB, Joyner MA, Gearhardt AN, Stewart JL, Paulus MP (2020) Mapping sign-tracking and goal-tracking onto human behaviors. Neurosci Biobehav Rev 111:84–94. 10.1016/j.neubiorev.2020.01.018 31972203 PMC8087151

[B20] Cope LM, Gheidi A, Martz ME, Duval ER, Khalil H, Allerton T, Morrow JD (2023) A mechanical task for measuring sign- and goal-tracking in humans: a proof-of-concept study. Behav Brain Res 436:114112. 10.1016/j.bbr.2022.114112 36115435 PMC10153473

[B21] Decker AL, Duncan K (2020) Acetylcholine and the complex interdependence of memory and attention. Curr Opin Behav Sci 32:21–28. 10.1016/j.cobeha.2020.01.013

[B22] Demeter E, Sarter M, Lustig C (2008) Rats and humans paying attention: cross-species task development for translational research. Neuropsychology 22:787–799. 10.1037/a0013712 18999353 PMC2705465

[B23] Demeter E, Guthrie SK, Taylor SF, Sarter M, Lustig C (2013) Increased distractor vulnerability but preserved vigilance in patients with schizophrenia: evidence from a translational Sustained Attention Task. Schizophr Res 144:136–141. 10.1016/j.schres.2013.01.003 23374860

[B24] Donaldson W (1992) Measuring recognition memory. J Exp Psychol Gen 121:275–277. 10.1037//0096-3445.121.3.275 1402701

[B25] Donovan E, Avila C, Klausner S, Parikh V, Fenollar-Ferrer C, Blakely RD, Sarter M (2022) Disrupted choline clearance and sustained acetylcholine release in vivo by a common choline transporter coding variant associated with poor attentional control in humans. J Neurosci 42:3426–3444. 10.1523/JNEUROSCI.1334-21.2022 35232764 PMC9034784

[B26] Duque A, Balatoni B, Detari L, Zaborszky L (2000) EEG correlation of the discharge properties of identified neurons in the basal forebrain. J Neurophysiol 84:1627–1635. 10.1152/jn.2000.84.3.1627 10980032

[B27] Echevarria DJ, Brewer A, Burk JA, Brown SN, Manuzon H, Robinson JK (2005) Construct validity of an operant signal detection task for rats. Behav Brain Res 157:283–290. 10.1016/j.bbr.2004.07.013 15639179

[B28] Ersche KD, Bullmore ET, Craig KJ, Shabbir SS, Abbott S, Müller U, Ooi C, Suckling J, Barnes A, Sahakian BJ, Merlo-Pich EV, Robbins TW (2010) Influence of compulsivity of drug abuse on dopaminergic modulation of attentional bias in stimulant dependence. Arch Gen Psychiatry 67:632–644. 10.1001/archgenpsychiatry.2010.60 20530013 PMC3664786

[B29] Ersche KD, Barnes A, Jones PS, Morein-Zamir S, Robbins TW, Bullmore ET (2011) Abnormal structure of frontostriatal brain systems is associated with aspects of impulsivity and compulsivity in cocaine dependence. Brain 134:2013–2024. 10.1093/brain/awr138 21690575 PMC3122375

[B30] Ersche KD, Jones PS, Williams GB, Turton AJ, Robbins TW, Bullmore ET (2012) Abnormal brain structure implicated in stimulant drug addiction. Science 335:601–604. 10.1126/science.1214463 22301321

[B31] Esterman M, Rothlein D (2019) Models of sustained attention. Curr Opin Psychol 29:174–180. 10.1016/j.copsyc.2019.03.005 30986621

[B32] Field M, Cox WM (2008) Attentional bias in addictive behaviors: a review of its development, causes, and consequences. Drug Alcohol Depend 97:1–20. 10.1016/j.drugalcdep.2008.03.030 18479844

[B33] Fitzpatrick CJ, Gopalakrishnan S, Cogan ES, Yager LM, Meyer PJ, Lovic V, Saunders BT, Parker CC, Gonzales NM, Aryee E, Flagel SB, Palmer AA, Robinson TE, Morrow JD (2013) Variation in the form of Pavlovian conditioned approach behavior among outbred male Sprague-Dawley rats from different vendors and colonies: sign-tracking vs. goal-tracking. PLoS One 8:e75042. 10.1371/journal.pone.0075042 24098363 PMC3787975

[B34] Flagel SB, Watson SJ, Robinson TE, Akil H (2007) Individual differences in the propensity to approach signals vs goals promote different adaptations in the dopamine system of rats. Psychopharmacology (Berl) 191:599–607. 10.1007/s00213-006-0535-8 16972103

[B35] Flagel SB, Akil H, Robinson TE (2009) Individual differences in the attribution of incentive salience to reward-related cues: implications for addiction. Neuropharmacology 56 [Suppl 1]:139–148. 10.1016/j.neuropharm.2008.06.027 18619474 PMC2635343

[B36] Gemperle AY, McAllister KH, Olpe HR (2003) Differential effects of iloperidone, clozapine, and haloperidol on working memory of rats in the delayed non-matching-to-position paradigm. Psychopharmacology (Berl) 169:354–364. 10.1007/s00213-003-1459-1 12827343

[B37] Gielow MR, Zaborszky L (2017) The input-output relationship of the cholinergic basal forebrain. Cell Rep 18:1817–1830. 10.1016/j.celrep.2017.01.060 28199851 PMC5725195

[B38] Gileta AF, Fitzpatrick CJ, Chitre AS, St Pierre CL, Joyce EV, Maguire RJ, McLeod AM, Gonzales NM, Williams AE, Morrow JD, Robinson TE, Flagel SB, Palmer AA (2022) Genetic characterization of outbred Sprague Dawley rats and utility for genome-wide association studies. PLoS Genet 18:e1010234. 10.1371/journal.pgen.1010234 35639796 PMC9187121

[B39] Goldbach HC, Akitake B, Leedy CE, Histed MH (2021) Performance in even a simple perceptual task depends on mouse secondary visual areas. Elife 10:e62156. 10.7554/eLife.62156 33522482 PMC7990500

[B40] Goldstein RZ, Volkow ND (2011) Dysfunction of the prefrontal cortex in addiction: neuroimaging findings and clinical implications. Nat Rev Neurosci 12:652–669. 10.1038/nrn3119 22011681 PMC3462342

[B41] Gomez JL, Bonaventura J, Lesniak W, Mathews WB, Sysa-Shah P, Rodriguez LA, Ellis RJ, Richie CT, Harvey BK, Dannals RF, Pomper MG, Bonci A, Michaelides M (2017) Chemogenetics revealed: DREADD occupancy and activation via converted clozapine. Science 357:503–507. 10.1126/science.aan2475 28774929 PMC7309169

[B42] Greenwald AG, Gonzalez R, Harris RJ, Guthrie D (1996) Effect sizes and p values: what should be reported and what should be replicated? Psychophysiology 33:175–183. 10.1111/j.1469-8986.1996.tb02121.x 8851245

[B43] Gritti I, Henny P, Galloni F, Mainville L, Mariotti M, Jones BE (2006) Stereological estimates of the basal forebrain cell population in the rat, including neurons containing choline acetyltransferase, glutamic acid decarboxylase or phosphate-activated glutaminase and colocalizing vesicular glutamate transporters. Neuroscience 143:1051–1064. 10.1016/j.neuroscience.2006.09.024 17084984 PMC1831828

[B44] Gritton HJ, Howe WM, Mallory CS, Hetrick VL, Berke JD, Sarter M (2016) Cortical cholinergic signaling controls the detection of cues. Proc Natl Acad Sci U S A 113:E1089–E1097. 10.1073/pnas.1516134113 26787867 PMC4776505

[B45] Gyengesi E, Andrews ZB, Paxinos G, Zaborszky L (2013) Distribution of secretagogin-containing neurons in the basal forebrain of mice, with special reference to the cholinergic corticopetal system. Brain Res Bull 94:1–8. 10.1016/j.brainresbull.2013.01.009 23376788 PMC3628291

[B46] Hassani OK, Lee MG, Henny P, Jones BE (2009) Discharge profiles of identified GABAergic in comparison to cholinergic and putative glutamatergic basal forebrain neurons across the sleep-wake cycle. J Neurosci 29:11828–11840. 10.1523/JNEUROSCI.1259-09.2009 19776269 PMC2790860

[B47] Head J, Helton WS (2014) Sustained attention failures are primarily due to sustained cognitive load not task monotony. Acta Psychol (Amst) 153:87–94. 10.1016/j.actpsy.2014.09.007 25310454

[B48] Hopfinger JB, Buonocore MH, Mangun GR (2000) The neural mechanisms of top-down attentional control. Nat Neurosci 3:284–291. 10.1038/72999 10700262

[B49] Howe WM, Berry AS, Francois J, Gilmour G, Carp JM, Tricklebank M, Lustig C, Sarter M (2013) Prefrontal cholinergic mechanisms instigating shifts from monitoring for cues to cue-guided performance: converging electrochemical and fMRI evidence from rats and humans. J Neurosci 33:8742–8752. 10.1523/JNEUROSCI.5809-12.2013 23678117 PMC3690786

[B50] Howe WM, Gritton HJ, Lusk NA, Roberts EA, Hetrick VL, Berke JD, Sarter M (2017) Acetylcholine release in prefrontal cortex promotes gamma oscillations and theta-gamma coupling during cue detection. J Neurosci 37:3215–3230. 10.1523/JNEUROSCI.2737-16.2017 28213446 PMC5373115

[B112] Huppé-Gourgues F, Jegouic K, Vaucher E (2018) Topographic organization of cholinergic innervation from the basal forebrain to the visual cortex in the rat. Front Neural Circuits 12:19. 10.3389/fncir.2018.00019 29662442 PMC5890115

[B51] Hwang E, Brown RE, Kocsis B, Kim T, McKenna JT, McNally JM, Han HB, Choi JH (2019) Optogenetic stimulation of basal forebrain parvalbumin neurons modulates the cortical topography of auditory steady-state responses. Brain Struct Funct 224:1505–1518. 10.1007/s00429-019-01845-5 30826928 PMC6532347

[B52] Kang JI, Groleau M, Dotigny F, Giguère H, Vaucher E (2014) Visual training paired with electrical stimulation of the basal forebrain improves orientation-selective visual acuity in the rat. Brain Struct Funct 219:1493–1507. 10.1007/s00429-013-0582-y 23700106

[B53] Kilts CD, Kennedy A, Elton AL, Tripathi SP, Young J, Cisler JM, James GA (2014) Individual differences in attentional bias associated with cocaine dependence are related to varying engagement of neural processing networks. Neuropsychopharmacology 39:1135–1147. 10.1038/npp.2013.314 24196947 PMC3957107

[B54] Kim K, Müller M, Bohnen NI, Sarter M, Lustig C (2019) The cortical cholinergic system contributes to the top-down control of distraction: evidence from patients with Parkinson’s disease. Neuroimage 190:107–117. 10.1016/j.neuroimage.2017.12.012 29277400 PMC6008164

[B55] Koshy Cherian A, Kucinski A, Pitchers KK, Yegla B, Parikh V, Kim Y, Valuskova P, Gurnani S, Lindsley CW, Blakely RD, Sarter M (2017) Unresponsive choline transporter as a trait neuromarker and a causal mediator of bottom-up attentional biases. J Neurosci 37:2947–2959. 10.1523/JNEUROSCI.3499-16.2017 28193693 PMC5354335

[B56] Kucinski A, Kim Y, Sarter M (2019) Basal forebrain chemogenetic inhibition disrupts the superior complex movement control of goal-tracking rats. Behav Neurosci 133:121–134. 10.1037/bne0000290 30688488 PMC6850517

[B57] Kurzban R, Duckworth A, Kable JW, Myers J (2013) An opportunity cost model of subjective effort and task performance. Behav Brain Sci 36:661–679. 10.1017/S0140525X12003196 24304775 PMC3856320

[B58] Lim J, Wu WC, Wang J, Detre JA, Dinges DF, Rao H (2010) Imaging brain fatigue from sustained mental workload: an ASL perfusion study of the time-on-task effect. Neuroimage 49:3426–3435. 10.1016/j.neuroimage.2009.11.020 19925871 PMC2830749

[B59] Lin SC, Nicolelis MA (2008) Neuronal ensemble bursting in the basal forebrain encodes salience irrespective of valence. Neuron 59:138–149. 10.1016/j.neuron.2008.04.031 18614035 PMC2697387

[B60] Lin SC, Gervasoni D, Nicolelis MA (2006) Fast modulation of prefrontal cortex activity by basal forebrain noncholinergic neuronal ensembles. J Neurophysiol 96:3209–3219. 10.1152/jn.00524.2006 16928796

[B61] Lovic V, Saunders BT, Yager LM, Robinson TE (2011) Rats prone to attribute incentive salience to reward cues are also prone to impulsive action. Behav Brain Res 223:255–261. 10.1016/j.bbr.2011.04.006 21507334 PMC3119757

[B62] Luck SJ, Ford JM, Sarter M, Lustig C (2012) CNTRICS final biomarker selection: control of attention. Schizophr Bull 38:53–61. 10.1093/schbul/sbr065 21765166 PMC3245597

[B63] Lustig C, Kozak R, Sarter M, Young JW, Robbins TW (2013) CNTRICS final animal model task selection: control of attention. Neurosci Biobehav Rev 37:2099–2110. 10.1016/j.neubiorev.2012.05.009 22683929 PMC3490036

[B64] Manns ID, Alonso A, Jones BE (2000) Discharge profiles of juxtacellularly labeled and immunohistochemically identified GABAergic basal forebrain neurons recorded in association with the electroencephalogram in anesthetized rats. J Neurosci 20:9252–9263. 10.1523/JNEUROSCI.20-24-09252.2000 11125003 PMC6773015

[B65] Marhe R, Luijten M, van de Wetering BJ, Smits M, Franken IH (2013) Individual differences in anterior cingulate activation associated with attentional bias predict cocaine use after treatment. Neuropsychopharmacology 38:1085–1093. 10.1038/npp.2013.7 23303067 PMC3629408

[B66] Martinez V, Sarter M (2008) Detection of the moderately beneficial cognitive effects of low-dose treatment with haloperidol or clozapine in an animal model of the attentional impairments of schizophrenia. Neuropsychopharmacology 33:2635–2647. 10.1038/sj.npp.1301661 18094665

[B67] McGaughy J, Sarter M (1995) Behavioral vigilance in rats: task validation and effects of age, amphetamine, and benzodiazepine receptor ligands. Psychopharmacology (Berl) 117:340–357. 10.1007/BF02246109 7770610

[B68] McGaughy J, Kaiser T, Sarter M (1996) Behavioral vigilance following infusions of 192 IgG-saporin into the basal forebrain: selectivity of the behavioral impairment and relation to cortical AChE-positive fiber density. Behav Neurosci 110:247–265. 10.1037//0735-7044.110.2.247 8731052

[B69] Meyer PJ, Lovic V, Saunders BT, Yager LM, Flagel SB, Morrow JD, Robinson TE (2012) Quantifying individual variation in the propensity to attribute incentive salience to reward cues. PLoS One 7:e38987. 10.1371/journal.pone.0038987 22761718 PMC3382216

[B70] Miesenbock G (2009) The optogenetic catechism. Science 326:395–399. 19833960 10.1126/science.1174520

[B71] Miller BT, D’Esposito M (2005) Searching for “the top” in top-down control. Neuron 48:535–538. 10.1016/j.neuron.2005.11.002 16301170

[B72] Miller EK, Cohen JD (2001) An integrative theory of prefrontal cortex function. Annu Rev Neurosci 24:167–202. 10.1146/annurev.neuro.24.1.167 11283309

[B73] Norman KJ, Koike H, McCraney SE, Garkun Y, Bateh J, Falk EN, Im S, Caro K, Demars MP, Morishita H (2021) Chemogenetic suppression of anterior cingulate cortical neurons projecting to the visual cortex disrupts attentional behavior in mice. Neuropsychopharmacol Rep 41:207–214. 10.1002/npr2.12176 33955711 PMC8340833

[B74] Paolone G, Angelakos CC, Meyer PJ, Robinson TE, Sarter M (2013) Cholinergic control over attention in rats prone to attribute incentive salience to reward cues. J Neurosci 33:8321–8335. 10.1523/JNEUROSCI.0709-13.2013 23658172 PMC3690461

[B75] Parada MA, Hernandez L, Puig de Parada M, Rada P, Murzi E (1997) Selective action of acute systemic clozapine on acetylcholine release in the rat prefrontal cortex by reference to the nucleus accumbens and striatum. J Pharmacol Exp Ther 281:582–588. 9103547

[B76] Parasuraman R, Mouloua M (1987) Interaction of signal discriminability and task type in vigilance decrement. Percept Psychophys 41:17–22. 10.3758/bf03208208 3822739

[B77] Parasuraman R, Warm JS, Dember WN (1987) Vigilance: taxonomy and utility. In: Ergonomics and human factors. Recent research in psychology (Mark LS, Warm JS, Huston JL, eds), pp 11–32. New York: Springer.

[B78] Parikh V, Kozak R, Martinez V, Sarter M (2007) Prefrontal acetylcholine release controls cue detection on multiple timescales. Neuron 56:141–154. 10.1016/j.neuron.2007.08.025 17920021 PMC2084212

[B79] Peterson VL, Richards JB, Meyer PJ, Cabrera-Rubio R, Tripi JA, King CP, Polesskaya O, Baud A, Chitre AS, Bastiaanssen TFS, Woods LS, Crispie F, Dinan TG, Cotter PD, Palmer AA, Cryan JF (2020) Sex-dependent associations between addiction-related behaviors and the microbiome in outbred rats. EBioMedicine 55:102769. 10.1016/j.ebiom.2020.102769 32403084 PMC7218262

[B80] Phillips KB, Sarter M (2020) Addiction vulnerability and the processing of significant cues: sign-, but not goal-, tracker perceptual sensitivity relies on cue salience. Behav Neurosci 134:133–143. 10.1037/bne0000353 31916796 PMC7078022

[B81] Pitchers KK, Flagel SB, O’Donnell EG, Woods LC, Sarter M, Robinson TE (2015) Individual variation in the propensity to attribute incentive salience to a food cue: influence of sex. Behav Brain Res 278:462–469. 10.1016/j.bbr.2014.10.036 25446811 PMC4382370

[B82] Pitchers KK, Phillips KB, Jones JL, Robinson TE, Sarter M (2017a) Diverse roads to relapse: a discriminative cue signaling cocaine availability is more effective in renewing cocaine seeking in goal trackers than sign trackers and depends on basal forebrain cholinergic activity. J Neurosci 37:7198–7208. 10.1523/JNEUROSCI.0990-17.2017 28659281 PMC5546399

[B83] Pitchers KK, Kane LF, Kim Y, Robinson TE, Sarter M (2017b) ‘Hot’ vs. ‘cold’ behavioural-cognitive styles: motivational-dopaminergic vs. cognitive-cholinergic processing of a Pavlovian cocaine cue in sign- and goal-tracking rats. Eur J Neurosci 46:2768–2781. 10.1111/ejn.13741 29044780 PMC6088792

[B84] Prasad D, Lustig C (2020) Correlated activation between striatal and cortical regions during a movement-related signal detection task: a re-analysis of two fMRI datasets. Impulse 17:1–14.

[B85] Robbins TW, Gillan CM, Smith DG, de Wit S, Ersche KD (2012) Neurocognitive endophenotypes of impulsivity and compulsivity: towards dimensional psychiatry. Trends Cogn Sci 16:81–91. 10.1016/j.tics.2011.11.009 22155014

[B86] Robinson TE, Yager LM, Cogan ES, Saunders BT (2014) On the motivational properties of reward cues: individual differences. Neuropharmacology 76 Pt B:450–459. 10.1016/j.neuropharm.2013.05.040 23748094 PMC3796005

[B87] Romens SE, Maccoon DG, Abramson LY, Pollak SD (2011) Cognitive style moderates attention to attribution-relevant stimuli. Cognit Ther Res 35:134–141. 10.1007/s10608-010-9345-8 21701701 PMC3119562

[B88] Rossi AF, Bichot NP, Desimone R, Ungerleider LG (2007) Top down attentional deficits in macaques with lesions of lateral prefrontal cortex. J Neurosci 27:11306–11314. 10.1523/JNEUROSCI.2939-07.2007 17942725 PMC6673036

[B89] Roth BL (2016) DREADDs for neuroscientists. Neuron 89:683–694. 10.1016/j.neuron.2016.01.040 26889809 PMC4759656

[B90] Rothman KJ (1990) No adjustments are needed for multiple comparisons. Epidemiology 1:43–46. 2081237

[B91] Rueter LE, Ballard ME, Gallagher KB, Basso AM, Curzon P, Kohlhaas KL (2004) Chronic low dose risperidone and clozapine alleviate positive but not negative symptoms in the rat neonatal ventral hippocampal lesion model of schizophrenia. Psychopharmacology (Berl) 176:312–319. 10.1007/s00213-004-1897-4 15179541

[B92] Sarter M (2015) Behavioral-cognitive targets for cholinergic enhancement. Curr Opin Behav Sci 4:22–26. 10.1016/j.cobeha.2015.01.004 28607947 PMC5466806

[B93] Sarter M, Bruno JP (2002) The neglected constituent of the basal forebrain corticopetal projection system: GABAergic projections. Eur J Neurosci 15:1867–1873. 10.1046/j.1460-9568.2002.02004.x 12099892

[B94] Sarter M, Fritschy JM (2008) Reporting statistical methods and statistical results in EJN. Eur J Neurosci 28:2363–2364. 10.1111/j.1460-9568.2008.06581.x 19087166

[B95] Sarter M, Phillips KB (2018) The neuroscience of cognitive-motivational styles: sign- and goal-trackers as animal models. Behav Neurosci 132:1–12. 10.1037/bne0000226 29355335 PMC5881169

[B96] Sarter M, Lustig C (2020) Forebrain cholinergic signaling: wired and phasic, not tonic, and causing behavior. J Neurosci 40:712–719. 10.1523/JNEUROSCI.1305-19.2019 31969489 PMC6975286

[B97] Sarter M, Lustig C, Blakely RD, Koshy Cherian A (2016) Cholinergic genetics of visual attention: human and mouse choline transporter capacity variants influence distractibility. J Physiol Paris 110:10–18. 10.1016/j.jphysparis.2016.07.001 27404793 PMC5164965

[B98] Saunders BT, Robinson TE (2010) A cocaine cue acts as an incentive stimulus in some but not others: implications for addiction. Biol Psychiatry 67:730–736. 10.1016/j.biopsych.2009.11.015 20045508 PMC2849872

[B99] Schad DJ, Rapp MA, Garbusow M, Nebe S, Sebold M, Obst E, Sommer C, Deserno L, Rabovsky M, Friedel E, Romanczuk-Seiferth N, Wittchen HU, Zimmermann US, Walter H, Sterzer P, Smolka MN, Schlagenhauf F, Heinz A, Dayan P, Huys QJM (2020) Dissociating neural learning signals in human sign- and goal-trackers. Nat Hum Behav 4:201–214. 10.1038/s41562-019-0765-5 31712764

[B100] Serences JT, Shomstein S, Leber AB, Golay X, Egeth HE, Yantis S (2005) Coordination of voluntary and stimulus-driven attentional control in human cortex. Psychol Sci 16:114–122. 10.1111/j.0956-7976.2005.00791.x 15686577

[B101] Sheynin Y, Rosa-Neto P, Hess RF, Vaucher E (2020) Cholinergic modulation of binocular vision. J Neurosci 40:5208–5213. 10.1523/JNEUROSCI.2484-19.2020 32457075 PMC7329301

[B102] Temple JG, Warm JS, Dember WN, Jones KS, LaGrange CM, Matthews G (2000) The effects of signal salience and caffeine on performance, workload, and stress in an abbreviated vigilance task. Hum Factors 42:183–194. 10.1518/001872000779656480 11022879

[B103] Thomson DR, Besner D, Smilek D (2015) A resource-control account of sustained attention: evidence from mind-wandering and vigilance paradigms. Perspect Psychol Sci 10:82–96. 10.1177/1745691614556681 25910383

[B104] Tingley D, Alexander AS, Quinn LK, Chiba AA, Nitz DA (2015) Cell assemblies of the basal forebrain. J Neurosci 35:2992–3000. 10.1523/JNEUROSCI.4432-14.2015 25698736 PMC6605588

[B105] Tingley D, Alexander AS, Quinn LK, Chiba AA, Nitz D (2018) Multiplexed oscillations and phase rate coding in the basal forebrain. Sci Adv 4:eaar3230. 10.1126/sciadv.aar3230 30083600 PMC6070333

[B106] Tomasi D, Goldstein RZ, Telang F, Maloney T, Alia-Klein N, Caparelli EC, Volkow ND (2007) Thalamo-cortical dysfunction in cocaine abusers: implications in attention and perception. Psychiatry Res 155:189–201. 10.1016/j.pscychresns.2007.03.002 17582746 PMC2265105

[B107] Tran FH, Spears SL, Ahn KJ, Eisch AJ, Yun S (2020) Does chronic systemic injection of the DREADD agonists clozapine-N-oxide or Compound 21 change behavior relevant to locomotion, exploration, anxiety, and depression in male non-DREADD-expressing mice? Neurosci Lett 739:135432. 10.1016/j.neulet.2020.135432 33080350

[B108] Volkow ND, Wang GJ, Ma Y, Fowler JS, Wong C, Ding Y-S, Hitzemann R, Swanson JM, Kalivas P (2005) Activation of orbital and medial prefrontal cortex by methylphenidate in cocaine-addicted subjects but not in controls: relevance to addiction. J Neurosci 25:3932–3939. 10.1523/JNEUROSCI.0433-05.2005 15829645 PMC6724925

[B109] Yager LM, Pitchers KK, Flagel SB, Robinson TE (2015) Individual variation in the motivational and neurobiological effects of an opioid cue. Neuropsychopharmacology 40:1269–1277. 10.1038/npp.2014.314 25425322 PMC4367472

[B110] Yang C, Thankachan S, McCarley RW, Brown RE (2017) The menagerie of the basal forebrain: how many (neural) species are there, what do they look like, how do they behave and who talks to whom? Curr Opin Neurobiol 44:159–166. 10.1016/j.conb.2017.05.004 28538168 PMC5525536

[B111] Zant JC, Kim T, Prokai L, Szarka S, McNally J, McKenna JT, Shukla C, Yang C, Kalinchuk AV, McCarley RW, Brown RE, Basheer R (2016) Cholinergic neurons in the basal forebrain promote wakefulness by actions on neighboring non-cholinergic neurons: an opto-dialysis study. J Neurosci 36:2057–2067. 10.1523/JNEUROSCI.3318-15.2016 26865627 PMC4748083

